# Molecular classification of human papilloma virus-negative head and neck squamous cell carcinomas: Cell cycle-based classifier and prognostic signature

**DOI:** 10.1371/journal.pone.0286414

**Published:** 2023-10-30

**Authors:** Hao Gu, Tingxuan Li, Narasimha M. Beeraka, Yufei Zheng, Xintan Zhang, Ruixia Song, Runze Zhou, Xiaoyan Wang, Olga Sukocheva, Ruitai Fan, Junqi Liu

**Affiliations:** 1 Department of Radiation Oncology & Cancer Center, The First Affiliated Hospital of Zhengzhou University, Zhengzhou, China; 2 Raghavendra Institute of Pharmaceutical Education and Research (RIPER), Anantapuramu, Andhra Pradesh, India; 3 Department of Human Anatomy, I.M. Sechenov First Moscow State Medical University (Sechenov University), Moscow, Russian Federation; 4 Herman B. Wells Center for Pediatric Research, Department of Pediatrics, Indiana University School of Medicine, Indianapolis, IN, United States of America; 5 Endocrinology Department, The First Affiliated Hospital of Zhengzhou University, Zhengzhou, China; 6 Discipline of Health Sciences, College of Nursing and Health Sciences, Flinders University, Bedford Park, South Australia, Australia; 7 Department of Hepatology, Royal Adelaide Hospital, SA Health, Adelaide, SA, Australia; King Faisal Specialist Hospital and Research Center, SAUDI ARABIA

## Abstract

The molecular classification of human papillomavirus (HPV)-negative head and neck squamous cell carcinomas (HNSCCs) remains questionable. Differentially expressed genes were detected between tumor and normal tissues and GSEA showed they are associated with cell cycle pathways. This study aimed to classify HPV-negative HNSCCs based on cell cycle-related genes. The established gene pattern was correlated with tumor progression, clinical prognosis, and drug treatment efficacy. Biological analysis was performed using HNSCC patient sample data obtained from the Cancer Genome Atlas (TCGA), Clinical Proteomic Tumor Analysis Consortium (CPTAC), and Gene Expression Omnibus (GEO) databases. All samples included in this study contained survival information. RNA sequencing data from 740 samples were used for the analysis. Previously characterized cell cycle-related genes were included for unsupervised consensus clustering. Two subtypes of HPV-negative HNSCCs (C1, C2) were identified. Subtype C1 displayed low cell cycle activity, ‘hot’ tumor microenvironment (TME), earlier N stage, lower pathological grade, better prognosis, and higher response rate to the immunotherapy and targeted therapy. Subtype C2 was associated with higher cell cycle activity, ‘cold’ TME, later N stage, higher pathological grade, worse prognosis, and lower response rate to the treatment. According to the nearest template prediction method, classification rules were established and verified. Our work explored the molecular mechanism of HPV-negative HNSCCs in the view of cell cycle and might provide new sights for personalized anti-cancer treatment.

## Introduction

The incidence of head and neck carcinomas (HNC) was ranked as sixth cancer-related worldwide, compared to the incidence of other tumors [1]. Approximately 90% of HNC patients are diagnosed with head and neck squamous cell carcinomas (HNSCCs) and among them, 60% of the carcinomas are considered locally advanced [2]. The implicit onset of carcinogenesis complicates an early diagnosis or prognosis, and HNSCCs often progress to the advanced stages with recurrent malignancies and metastasis [3]. HPV-positive HNSCCs can be differentiated from HPV-negative HNSCCs with specific gene expression profiles, mutations, and immunity phenotypes. This study investigated molecular mechanisms and markers of HPV-negative type of HNSCCs, which is accompanied by a higher incidence rate, and worse prognosis when compared to HPV-positive tumors [4]. HNSCC is a heterogenic disease, and several attempts have been made to classify it [5–8].

The therapeutic regimen for HPV-negative HNSCC is multimodal and commonly includes surgical intervention followed by chemo-radiotherapy. Cetuximab, and anti-EGFR monoclonal antibody-based therapeutic molecules are seldom preferred modalities as a treatment; sometimes, these are administered in a combination regimen with radiotherapy in patients with HPV-negative HNSCC. FDA-approved immune checkpoint inhibitors, including pembrolizumab and nivolumab, are often prescribed for the treatment of metastatic HNSCC [9]. Moreover, pembrolizumab is used for the treatment of unresectable malignancies [9–11]. However, the incomplete response to these regimens warrants the development of better treatment methods. To design better therapeutic strategies and enhance the overall survival of patients with HNSCC, it is necessary to identify the molecular and genetic HNSCC landscapes and therapeutic targets.

A recent proteogenomic investigation [12] classified HPV-negative HNSCCs into three subtypes as follows: HNSCC with chromosome instability (CIN), basal (elevation of several basal factors), and immune (activated immune signaling pathways) subtypes. CIN HNSCC subtype is marked by cell cycle activation (higher Cyclin-dependent kinases (CDK) 4/6 activity), recurrent genetic alterations (increased cyclin D1 (CCND1) and cyclin-dependent kinase inhibitor 2a (CDKN2A) expression), and the worst prognosis [11, 13].

Dysregulated cell cycle function and uncontrolled cell proliferation are typical characteristics of malignant cells [14]. CDK is one of the main regulators of the cell cycle [15], which can be selectively inhibited [15]. Different cyclin-CDK complexes are activated and phosphorylate their target proteins during specific phases of the cell cycle. The cell cycle is a 4-stage process consisting of gap 1 (G1), synthesis (S), gap 2 (G2), and mitosis (M) phase. The cyclin-CDK 4/6 complex is involved in the initiation of the G1/S phase transition [16–20]. Previous preclinical studies indicated the efficacy of systemic CDK4/CDK6 inhibition, which induced immunomodulatory effects and boosted antitumor immunity [21, 22]. A complex regimen of CDK4/6 inhibitors including, palbociclib, ribociclib, and abemaciclib increased breast cancer sensitivity to the applied immunotherapy [23].

Several clinical trials [24–26] addressed the effect of CDK4/6 inhibition in drug-resistant and refractory HPV-negative HNSCCs. Cell cycle-associated pathways were shown to have a prominent role in HPV-negative HNSCCs [27]. Considering the key role of cell cycle regulation in the development of metastatic HNSCCs, we hypothesized that HNSCCs can be grouped according to distinct molecular subtypes and the activity of cell cycle-related genes. Moreover, mutational alterations in the tumor suppressor genes (TSG) represent a significant challenge for successful HNSCC treatment. TSG mutations may reflect the molecular pathology of HNSCC. TSG pattern-based model was suggested for the development of therapeutic strategies [27]. The gene set enrichment analysis (GSEA) indicated abnormal expression levels of cell cycle-associated gene sets, including E2F targets, MYC, and G2M checkpoint [27].

Accordingly, we identified the differentially expressed genes (DEGs) in the paired normal and tumor samples collected through the GSE142083 dataset. Multiple independent HNSCC cohorts (from different collection centers) were analyzed. The unsupervised clustering analysis of cell cycle-associated genes has identified two HNSCC subtypes: cluster 1 and cluster 2 (C1 and C2). Subsequently, we compared several crucial characteristics of C1 and C2 subtypes, including the overall survival, cell cycle signature enrichment scores, clinical stages, immune infiltration, and drug sensitivity.

## Materials and methods

### Acquisition of HNSCCS dataset source & preliminary analysis

**Fig 1:**
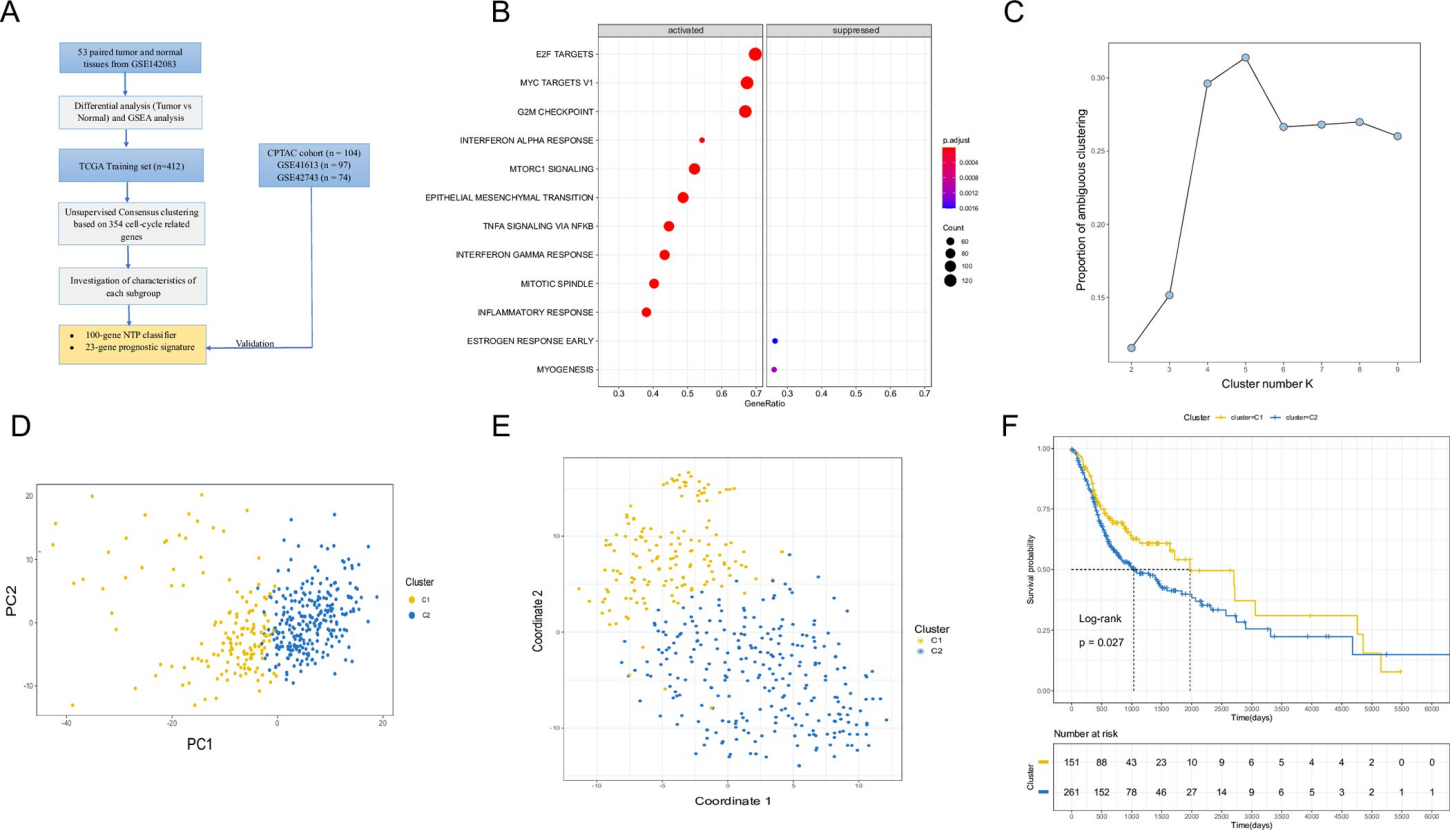
Identification of HPV-negative HNSCC subtypes using unsupervised consensus clustering in the TCGA cohort. (A). Flow chart of this study design. (B). Dotplot of GSEA based on differentially expressed genes between tumor tissues and normal tissues in GSE142083. (C). Proportion of ambiguous clustering (PAC) was estimated. A low value of PCA implies a flat middle segment, allowing conjecture of the optimal k (k = 2) by the lowest PAC. (D). PCA was based on the expression of 354 survival-related cell cycle genes in C1 and C2. The yellow dots indicate C1; blue dots indicate C2. (E). T-SNE analysis depicted the stratification of two subtypes. (F). Kaplan-Meier analysis for the overall survival rates in C1 and C2 subtypes (TCGA cohort; P<0.05).

The adapted workflow for the study stages is presented in **Fig 1A**. Gene-expression data and clinical information were retrieved from the TCGA database, Clinical Proteomic Tumor Analysis Consortium (CPTAC) [12], and Gene-Expression Omnibus (GEO) database. Consequently, five eligible HNSCC cohorts were included (TCGA-HNSCC, CPTAC-HNSCC, GSE42743, GSE41613, and GSE142083) and analyzed. The TCGA data (regarding Fragments per kilobase of transcript per million mapped fragments (FPKM), mutation) was downloaded and assessed using the R package ‘TCGAbiolinks’ [28]. The collected data was transformed to indicate transcripts per kilobase million (TPM) values for the high-throughput sequencing datasets (TCGA-HNSCC, CPTAC-HNSCC, and GSE142083). GSE42743 and GSE41613 were merged into a GPL570 cohort. The two normalized matrix files were directly downloaded from the GEO website. The R package *‘*hgu133plus2’ was used to convert the probe id to the gene symbol. For multiple probes corresponding to one gene symbol, the probe with the highest average value was chosen to represent the gene value. There were 20,161 common genes in both microarray datasets. Next, the combat function in the ‘SVA’ R package was applied to remove the batch effects and merge two datasets. The GSE142083 dataset was used to perform differential gene analysis and GSEA. The GSE142083 dataset was not used for the prognosis model due to insufficient overall survival information. The baseline information for the eligible HNSCC datasets is shown in **Table 1**. Subsequently, the data was assessed using R (version 4.1.2) and R-Bioconductor packages.

**Table 1:** Clinical parameters for 687 HPV-negative HNSCC patients.

Variable	TCGA-HNSCCs (N = 412)	CPTAC-HNSCCs (N = 104)	GSE41613 (N = 97)	GSE42743 (N = 74)
**Age**				
**<60**	**243**	**63**	**50**	**37**
**≥60**	**169**	**41**	**47**	**37**
**Gender**				
**Male**	**287**	**91**	**66**	**58**
**Female**	**125**	**13**	**31**	**16**
**Histologic grade**				
** G1 Well differentiated**	**58**	**18**	**NA**	**NA**
** G2 Moderately differentiated**	**258**	**75**	**NA**	**NA**
** G3 Poorly differentiated**	**87**	**11**	**NA**	**NA**
** NA**	**9**			
**Tumor stage**				
** Stage I**	**25**	**7**	**41**	**3**
** Stage II**	**58**	**25**		**16**
** Stage III**	**66**	**27**	**56**	**15**
** Stage IV**	**224**	**45**		**40**
** NA**	**39**			

### Transcriptome differential gene analysis and GSEA

In this study, a total of 53 paired laryngeal squamous cell carcinomas and adjacent normal tissues from the GSE142083 dataset were selected. An annotated gene set file (h.all.v7.2.symbols.gmt) was chosen as a reference. GSEA was conducted using the R package ‘ClusterProfiler’[29]. The threshold was set at P < 0.05.

### Unsupervised consensus clustering for cell cycle-regulating genes

Data related to 1875 genes involved in the cell cycle regulation was acquired from MSigDB (https://www.gsea-msigdb.org/gsea/index.jsp). Two gene sets, KEGGCELLCYCLE and GOCELLCYCLE, were retrieved and considered as cell cycle-related genesets. A filtering procedure was conducted prior to executing the consensus clustering analysis. Univariate Cox regression analysis was adopted to evaluate the association of the collected gene sets (TCGA and CPTAC) with overall survival (OS) using the R package ‘survival’. Subsequently, a total of 354 genes with significant (P < 0.05) influence over prognosis were chosen for sample clustering **(S2 Table)**. According to Euclidean and Ward’s linkage, the “Km” method was employed during this analysis. ‘ConsensusClusterPlus’ R package was used to perform clustering analysis. The simulation was set to 1,000 repeats to confirm the stability within the classification. The total number of clusters was set to the range from 2 to 9. The optimal number was assessed by examining the cumulative distribution function (CDF) of the consensus score and the proportion of ambiguous clustering (PAC). ‘Nbclust’ package was adopted for the consequent validation of the optimal number. Principal component analysis (PCA) and t-distributed stochastic neighbor embedding (t-SNE) were employed to verify the reliability of consensus clusters based on the above genes. Overall survival curves were constructed to analyze the prognosis using the Kaplan-Meier method and log-rank test.

### Generation and validation of the classifier

DEGs were determined during the comparison of two HNSCC subtypes using the ‘limma’ package. In each subtype, the top 50 genes (with the largest log2FC value, adjusted P value < 0.05) were chosen. Therefore, a 100-gene classifier was built. The nearest template prediction (NTP) analysis was performed in the TCGA cohort. Prediction results were compared to the original cluster. Then, the same prediction was repeated in the CPTAC-cohort and GPL570 combined cohort (including GSE41613 and GSE42743). The cell-cycle-related genes set enrich scores and clinical characteristics were compared.

### Classification of HNSCC subtypes into C1 and C2 subtypes

The cell cycle-associated gene sets that were statistically significant in the previous GSEA analysis were included and identified as reference gene sets for ssGSEA (single-sample gene-set enrichment analysis) to analyze the degree of enrichment of cell cycle signatures among the two molecular subtypes. Clinical characteristics including pathological grade and tumor stages were compared between the two subtypes. For both the molecular subtypes, we subsequently estimated the tumor microenvironment (TME) signature and tested the difference between the two clusters. Furthermore, TME, tumor-metabolism-related, and tumor-intrinsic signatures were estimated and compared among two subtypes.

### Estimation of immune infiltration into tumor tissues

The ‘estimate’[30] method was adopted to quantify the overall infiltration of immune cells and stromal cells in tumor tissues. Two gene signatures related to clinical response to PD-1 blockade were estimated according to the previously published reports [31]. We used the ‘cibersort’ [32] algorithm to quantify the 22 relative abundance of cell infiltration in TCGA-cohort. The chosen cells included CD4 and CD8 T cells, B cells, macrophages, regulatory T cells, and others.

### Potential benefits of using each cluster subtype in immunotherapy and cancer-targeted therapy

The tumor immune dysfunction and exclusion (TIDE) algorithm [33] was used to define individual responses to immunotherapy by immune checkpoint anti-PD-1 therapy. Subclass mapping [34] method (using GenePattern) was adopted to assess the similarity between samples collected in the TCGA-HNSCCs cohort and two other clinical treatment cohorts, including 27 SKCM samples (patients/cohort received anti-PD1-therapy [35]) and 28 HNSCCs samples (patients/cohort received cetuximab (anti-EGFR antibody)) therapy [36].

### Data sample with somatic alterations

The mutation data relevant to the TCGA-HNSCCs cohort was downloaded using ‘TCGAbiolinks’. The top 20 driver genes accompanied by the highest mutation frequency in two subtypes were identified using the R package ‘maftools’. Fisher exact test was employed to compare differences in the number of mutations between C1 and C2 subtypes. MutSigCV method was applied to assess significant mutated genes (SMGs) across the HPV-negative HNSCC samples with default parameters. The threshold was set as *q* < 0.05 [37].

### Development and validation of a prognostic signature

We established a prognosis signature named Cyclescore. DEGs between C1 and C2 subtypes were identified primarily (statistically significant differences, P < 0.05). Following this, univariate Cox proportional hazards regression was performed for DEGs in TCGA and CPTAC cohorts respectively (P < 0.05). The intersection genes were selected to build the prognosis signature. The TCGA cohort of 412 cases was randomized into training subsets and internal validation subsets using ‘caret’ package based on a 5-fold cross-validation method of sampling. The TCGA training subset included 4-fold cross-validation with HNSCCs samples, and the internal validation subsets included the remaining samples. Cox proportional hazards regression with 10-fold cross-validation was used to generate the most reliable prognostic gene model using ‘glmnet’ package [38]. The corresponding lasso coefficients were derived from the TCGA training subset and employed to validate the internal and external cohorts. The risk score was calculated using the formula: $\Sigma_{i=1}^{n}$coef (i)* (expression (i)). The median risk score of the TCGA training subset was considered as a uniform cutoff and validated in other cohorts. The cutoff value was determined after establishing the signature; subsequently, CPTAC and GPL570 cohorts were identified as external validation cohorts. Gene expressions in each cohort were completely standardized (mean value: 0, SD: 1) with R function ‘scale’. This transformation could increase the comparability of gene data across platforms.

### Statistical analysis

All data were analyzed using R software (https://www.r-project.org/). Wilcoxon test and Fisher’s exact test were adapted to compare the categorical and continuous variables in C1 and C2 subtypes, respectively. The log-rank test and Kaplan-Meier curve were used for the survival analysis. Differences were considered to be significant at P-value< 0.05.

## Results

### Differentially expressed genes between tumor and normal tissues and GSEA analysis

Dysregulated gene sets were identified during the comparison of GSE142083 between 53 laryngeal HNSCCs and 53 paired adjacent normal mucosa tissues. Consequently, the differentially expressed genes (DEGs) were identified. GSEA was conducted to establish the functional enrichment of DEGs as previously described [39, 40]. The GSEA plot demonstrates that cell cycle-regulating pathways, including E2F, MYC targets V1 and G2M checkpoint are activated in high ranks (**Fig 1B–1D**). Epithelial-mesenchymal transition (EMT), interferon (IFN)-α and IFN-γ response was also highly ranked.

### Unsupervised clustering identifies two subtypes

Univariate cox regression analysis was used to screen genes with survival significance in cell cycle-related genes (P < 0.05). We found that 354 genes were significantly linked to survival in both TCGA and CPTAC cohorts. These genes were used for the sample clustering. K = 2 was chosen as the optimal number of clusters considering the maximum consensus rate and minimum ambiguity rate (**S1A, S1B Fig**). The ‘NbClust’ method also confirmed the optimal number of clusters (**S1A–S1C Fig**). PCA analysis and t-distributed stochastic neighbor embedding (t-SNE) methods validated the subtype grouping according to the expression matrix of survival-related cell cycle regulating genes (**Fig 1D–1E**). Comparative overall survival (k = 2) for subtypes C1 and C2 is shown in **Fig 1F**.

### The differences between clinical and molecular characteristics in two subtypes and the performance of the NTP classifier

**Fig 2:**
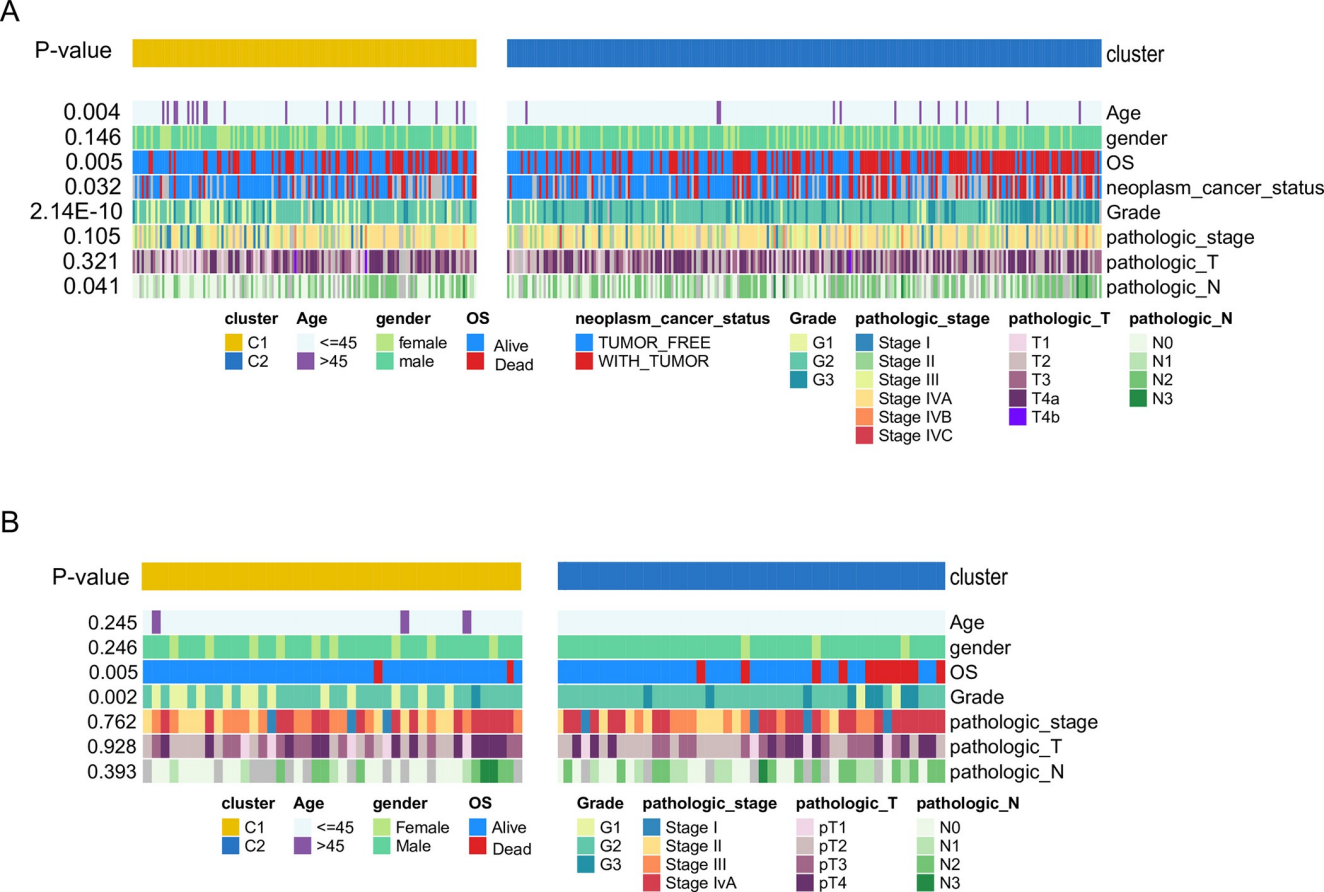
Comparison of clinical characteristics pertinent to C1 and C2 subtypes, (A) TCGA cohort, (B) CPTAC cohort.

C1 and C2 subtypes identified in the TCGA cohort were further compared using the NTP approach. This classifier was used to predict molecular HNSCC subtypes in TCGA-HNSCCs, CPTAC, and GPL570 cohorts. The earlier tumor stages, lower pathological grade, better OS status, tumor-free status, and older age were observed in C1, compared to C2 (**Fig 2A**). In the CPTAC test set, OS status and grade, two crucial clinical features, still have obvious differences in NTP-predicted subgroups (**Fig 2B**). In both TCGA and CPTAC cohorts, the incompleteness of clinical data has a huge negative impact on the observation of clinical parameters (**S1 Table**).

**Fig 3:**
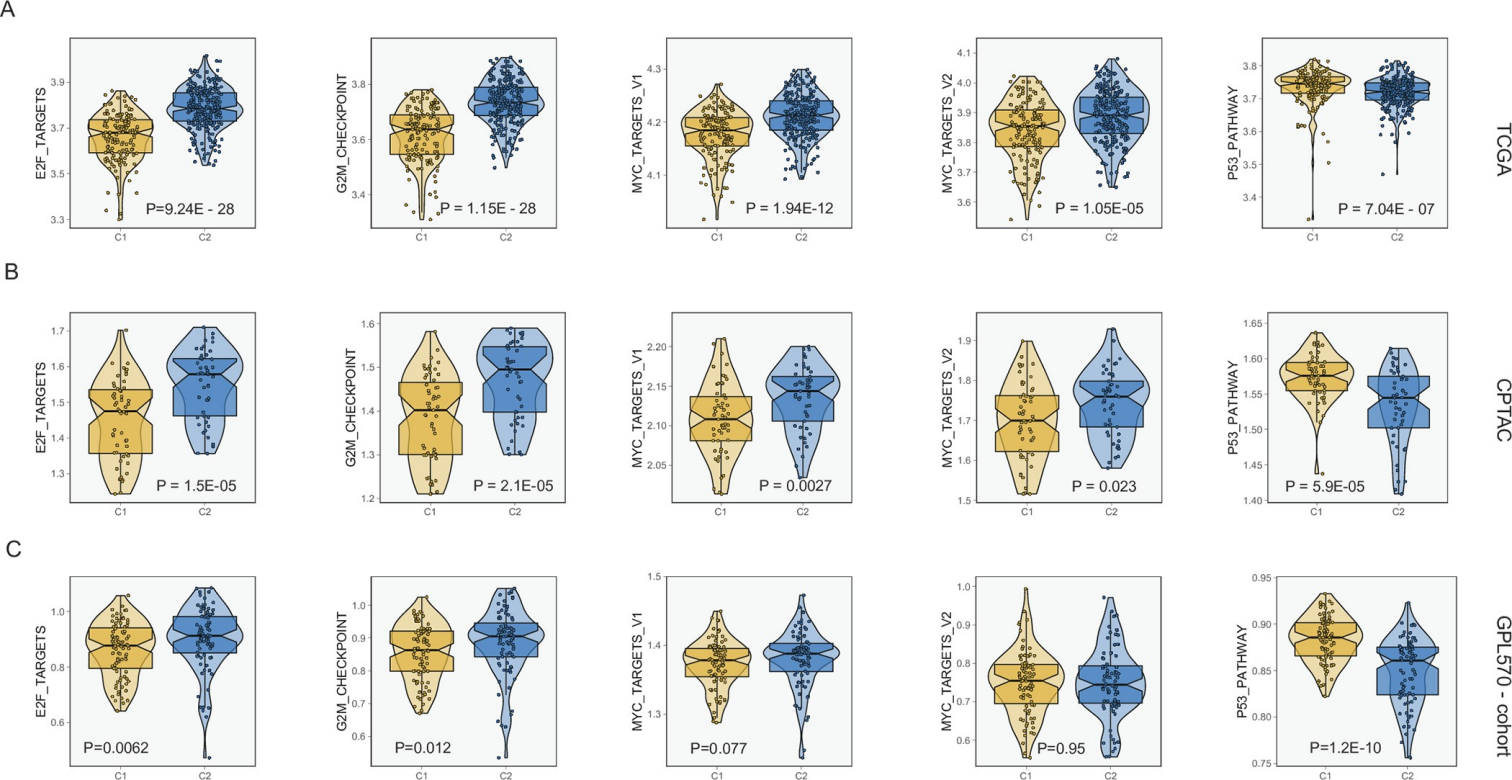
**(A), (B), (C)**: Comparison of ssGSEA scores of cell cycle pathways (E2F targets, G2M checkpoint, MYC targets, p53 pathway) between C1 and C2 subtypes in different data sets (TCGA, CPTAC, and GPL570 combined cohort).

Considering that this study is focused on cell cycle activity, we compared multiple cell cycle-related pathways between C1 and C2 subtypes using the box plot. E2F, G2M checkpoint, MYC, and P53 pathway enrichment scores were estimated for C1 and C2 subtypes using ssGSEA in different cohorts (TCGA, CPTAC, and GPL570 combined cohort). Wilcoxon test confirmed that the C2 subtype was marked by a higher cell cycle-related pathway enrichment score than C1. The analysis was conducted through multiple independent cohorts. The exception was the comparison of MYC targets v2 was not found to be significantly different in the GPL570 cohort (**Fig 3**). The observed differences were consistent with clinicopathological characteristics and proved the reliability of the derived NTP classifier for the analysis of molecular mechanisms.

**Fig 4:**
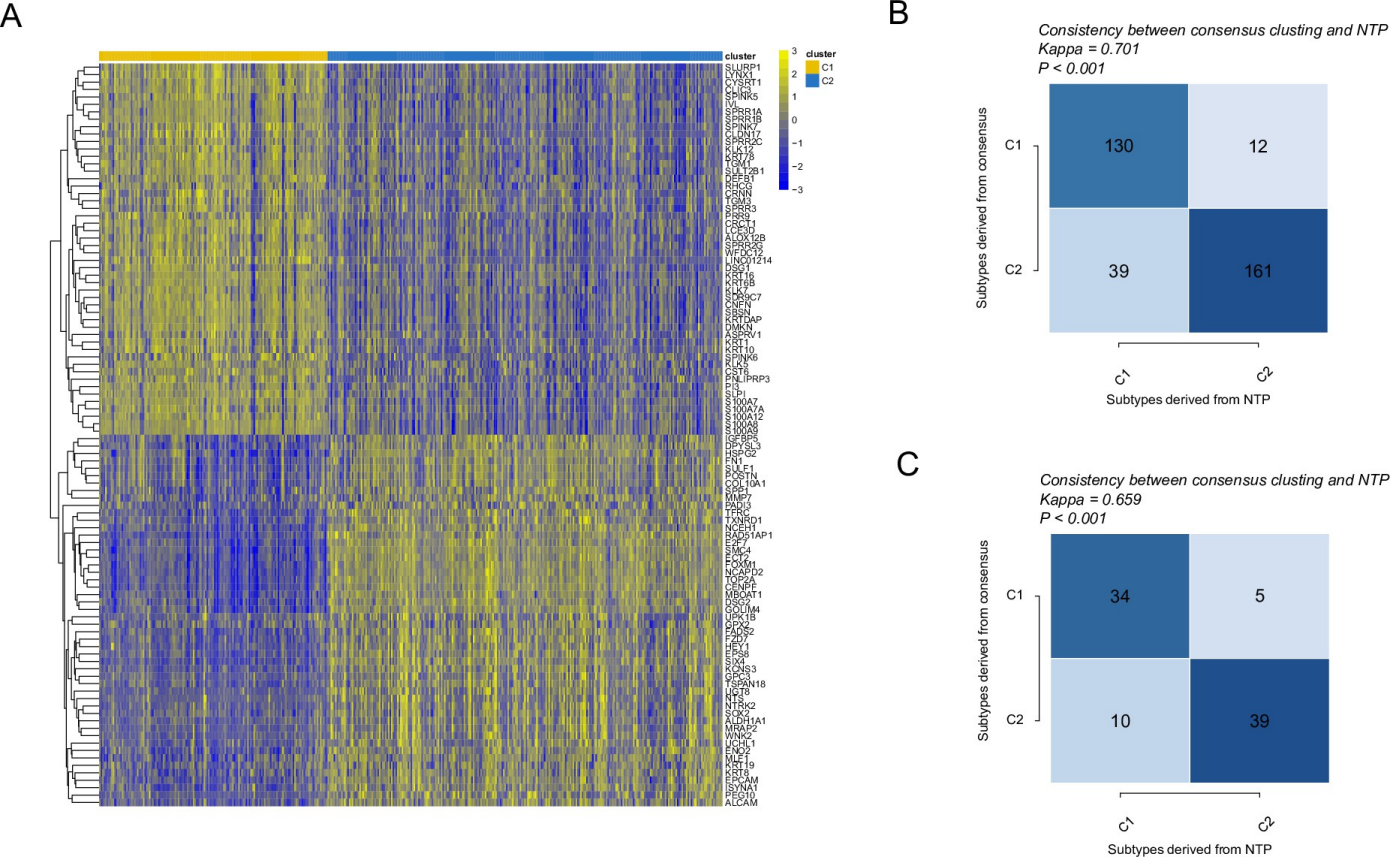
Performance and validation of the classifier. **(A)**. Heatmap of gene expression profiles involved in NTP classifier. **(B-C)**. Performance of the classifier (B, TCGA training cohort; bottom, CPTAC test cohort). The vertical axis shows the subtype resulting from the clustering result; the horizontal axis shows the subtype predicted by NTP.

After comprehensive consideration of the prediction accuracy, we selected the top 50 genes with the highest fold change in C1 and C2 and generated the NTP classifier (**Fig 4A, S3 Table**). Then, the NTP classifier’s accuracy was evaluated in TCGA and CPTAC cohorts separately **(Fig 4B)**. The prediction exhibited good concordance in the TCGA cohort (kappa = 0.701, P < 0.001) and medium agreement in the CPTAC cohort (kappa = 0.659, P < 0.001, **Fig 4C**).

### Differences in the mutation landscape between two subtypes

We assessed the mutation profiles of the C1 and C2 subtypes. The top 20 highest mutation frequencies in C1 and C2 subtypes were shown using *oncoplot* (**S3A, S3B Fig**). C2 demonstrated a higher mutation frequency than C1. The mutation frequencies of TP53, DNAH5, NSD1, RYR2, and CASP8 were significantly different between the two subtypes (Fisher exact test P value < 0.05; **Table 2**). MutSigCV identified 23 significantly mutated genes (SMGs, q < 0.05) for the samples with available mutation data (**S3C Fig**).

**Table 2:** Mutation characteristics of C1 and C2 subtypes in TCGA cohort.

Gene	C1 (%)	C2 (%)	P value
**TP53**	105 (70)	215 (83)	0.0037
**TTN**	50 (34)	111 (43)	0.0734
**FAT1**	44 (30)	62 (24)	0.2419
**CDKN2A**	40 (27)	55 (21)	0.2245
**MUC16**	21 (14)	55 (21)	0.0859
**CSMD3**	23 (15)	55 (21)	0.1532
**NOTCH1**	34 (23)	41 (16)	0.0862
**PIK3CA**	24 (16)	38 (15)	0.7749
**SYNE1**	21 (14)	51 (20)	0.1777
**LRP1B**	21 (14)	48 (19)	0.2740
**KMT2D**	18 (12)	46 (18)	0.1572
**PCLO**	21 (14)	46 (18)	0.4053
**DNAH5**	15 (10)	46 (18)	0.0430
**FLG**	21 (14)	33 (13)	0.7622
**USH2A**	18 (12)	39 (15)	0.4596
**NSD1**	7 (5)	40 (16)	0.0007
**RYR2**	9 (6)	37 (14)	0.0140
**CASP8**	27 (18)	23 (9)	0.0078
**PKHD1L1**	13 (9)	34 (13)	0.1999
**SI**	12 (8)	32 (12)	0.1889

### Different composition of TME and response rate to immunotherapy/targeted therapy between two subtypes

**Fig 5:**
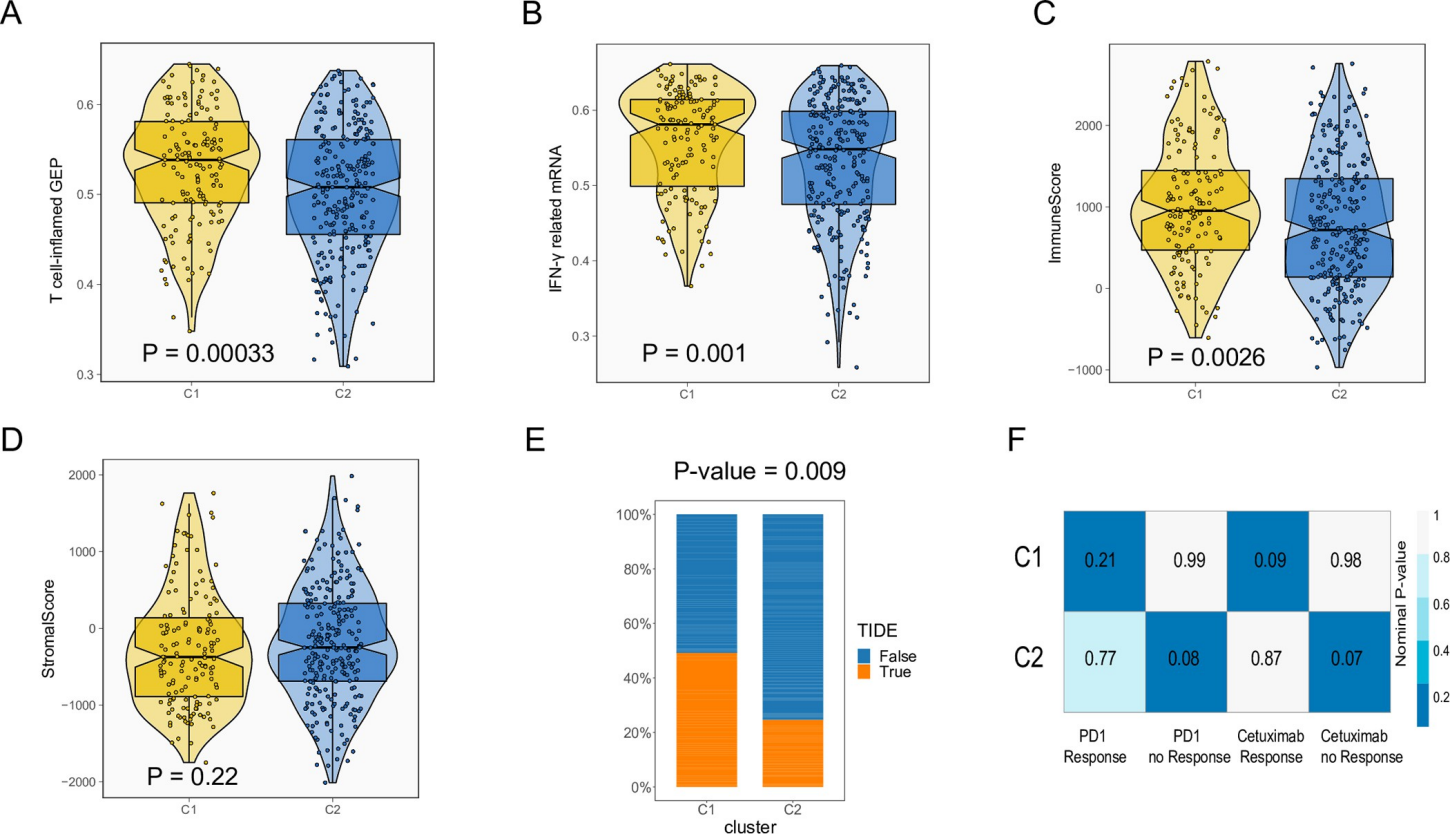
Assessment of immune-related signatures and drug responses in C1 and C2 subgroups in TCGA cohort. (**A, B**). Comparison of two immunotherapy-related gene signatures: T cell-inflamed gene expression and IFN-γ–related mRNA. (**C, D**) Assessment of immune and stromal cells’ population in tumor tissue among two subtypes. (**E**). The response rate to immunotherapy was estimated using the TIDE algorithm (Wilcoxon test, P = 0.009). (**F**). Submap analysis indicates that C1 is highly sensitive to anti-EGFR therapy (P = 0.09); while C2 is more likely to be unresponsive to immunotherapy (P = 0.08).

Increased and dysregulated cell cycle activity in cancer cells correlated with impaired anti-tumor immunity [41]. In this study, we compared C1 and C2-related immune landscapes. The abundance of 22 immune cells was assessed and compared using the *cibersort* method **(S4 Fig)**. The abundance of several anti-tumor immune effectors, including CD8/CD4 T-cells, activated NK-cells, and M1 macrophages were significantly higher in C1, compared to C2. We evaluated genomic biomarkers that may predict the efficacy of anti-PD1 therapy. Our analysis included T cell-inflamed gene expression profile (GEP) and IFN-γ–related mRNA profile. C1 subtype exhibited higher levels of T-cell inflamed GEP and IFN-γ–related mRNAs, compared to C2 **(Fig 5A and 5B)**. Later, the abundance of 22 immune cells was assessed and compared using the *cibersort* method. The abundance of several anti-tumor immune effectors, including CD8/CD4 T-cells, activated NK-cells, and M1 macrophages were significantly higher in C1, compared to C2 **(S4 Fig)**. A significant difference in immune score was observed among the two subgroups, with a higher immune score of C1 than C2 (P = 0.0026) (**Fig 5C**). There was no significant difference between the two subtypes of stromal score (**Fig 5D**).

The sensitivity of the two subtypes to the immunotherapy/targeted therapy was also compared. TIDE analysis showed that C1 cluster-linked patients are more likely to respond to the immunotherapy, compared to C2 subtype (P = 0.009) (**Fig 5E**). According to the submap result from the GenePattern website, C2 was correlated to the ‘no anti-PD1 response group’ (P = 0.08). As for cetuximab, the genomic features of C1 were similar to the ‘response’ patients (P = 0.09), while C2 was correlated to ‘no-response’ patients (P = 0.07) (**Fig 5F**).

### Generation of prognostic gene signature and related biological processes

**Fig 6:**
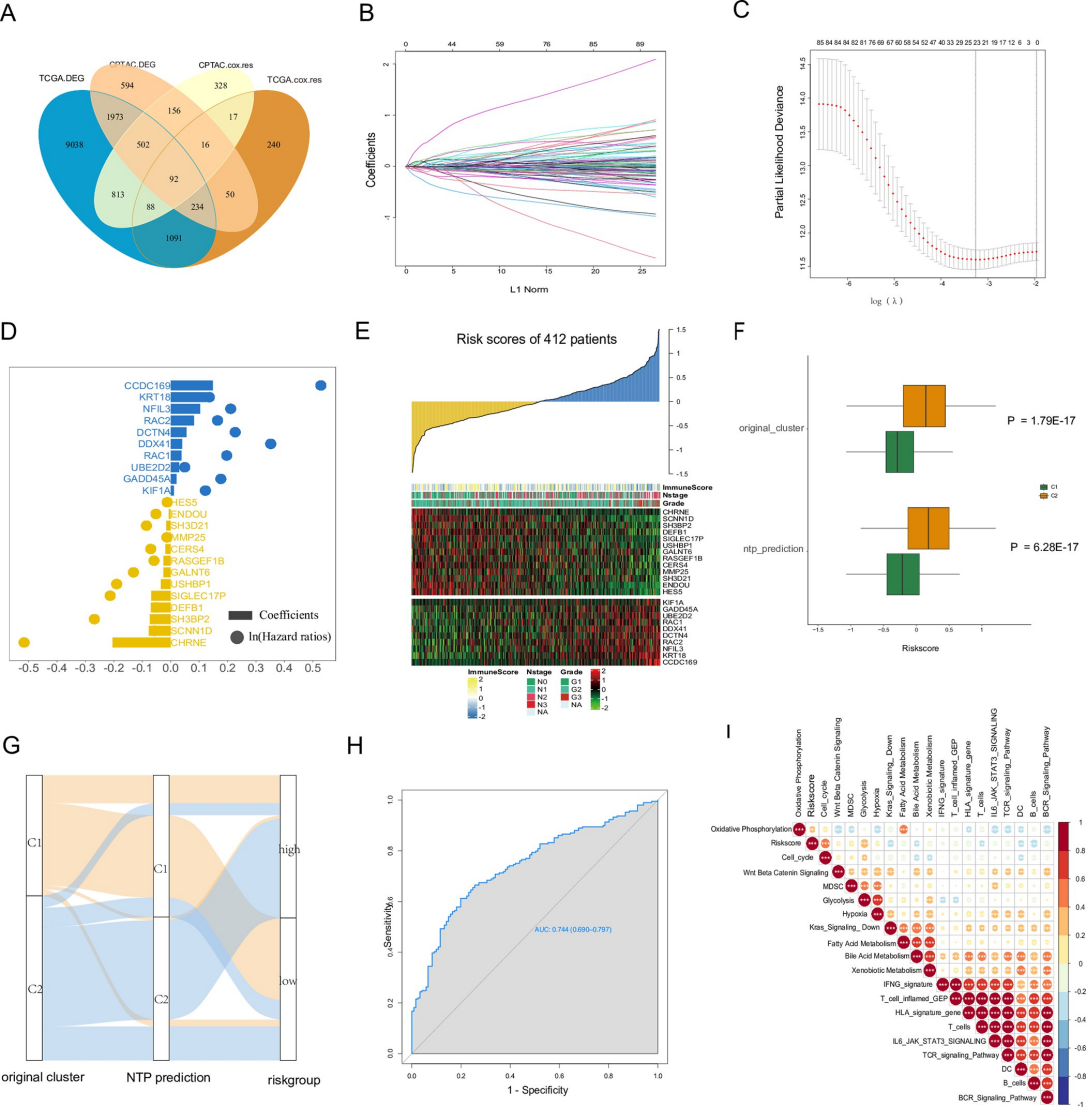
Development of prognostic gene signature in TCGA cohort. (**A**). Venn diagram of DEGs and prognostic-relevant associated genes in TCGA and CPTAC cohort. (**B**). Lasso coefficient profiles 92 subtype-related genes in the TCGA training cohort. (**C**). Partial likelihood deviance of 92 selected genes. (**D**). Cox regression analysis with lasso penalty reported the 23 prognosis-related genes, which were used to develop a prognostic signature (Cyclescore). Blue dots represent risk-related genes; yellow dots indicate protection-related genes. Corresponding coefficients from lasso and hazard ratios (multivariate Cox regression) are shown as horizontal bars and dots. (**E**). The expression profiles of 23 genes (included in the signature) are shown as a heatmap and ranked using Cyclescore in ascending order. Immune score (predicted by the ‘estimate’ package), pathological grade, and lymph node stage were annotated above the heatmap. (**F**) Expression pattern of clusters in Cyclescore in TCGA. (G) Sankey plot of the relationship between original clusters, the NTP prediction, and risk group. (**H**). The consistency between Cyclescore and the original cluster was estimated using a ROC curve. (**I**). The association between Cyclescore and 19 known cancer-related signatures is presented.

Common for both TCGA and CPTAC cohorts, 92 genes were engaged to build Cyclescore (**Fig 6A**). In the TCGA-training subset, least absolute shrinkage and selection operator (lasso) regression was adopted to acquire the best combination and select 23 genes, which were used to develop the signature. The lasso coefficients were shown in (**Fig 6B–6D**) and **S4 Table**. All the samples were segregated into high and low-risk groups using the uniform cutoff value (-0.0267). Following this, we collated all TCGA cohort samples and found that almost all 23 lasso-selected genes were expressed differently between the two risk groups. Expression pattern of clusters in Cyclescore in TCGA. Fig 6F showed that Cyclescore is significantly different in TCGA original clusters and NTP predictions, which not only proved the correlation between Cyclescore and our research theme: cell cycle, but also proved the reliability of NTP prediction. The interconnection among the original clusters, NTP predictions, and risk group is illustrated in the Sankey plot **Fig 6G**. The ROC curve confirmed that Cyclescore also served as a sensitive marker for cell cycle subtypes (**Fig 6H**). Total of 19 known intracellular and extracellular signatures were extracted using package ‘IOBR’ [42]. The correlation between Cyclescore and these biomarkers are shown in **Fig 6I**.

### Verification of Cyclescore and the uniform cutoff value for multiple cohorts

**Fig 7:**
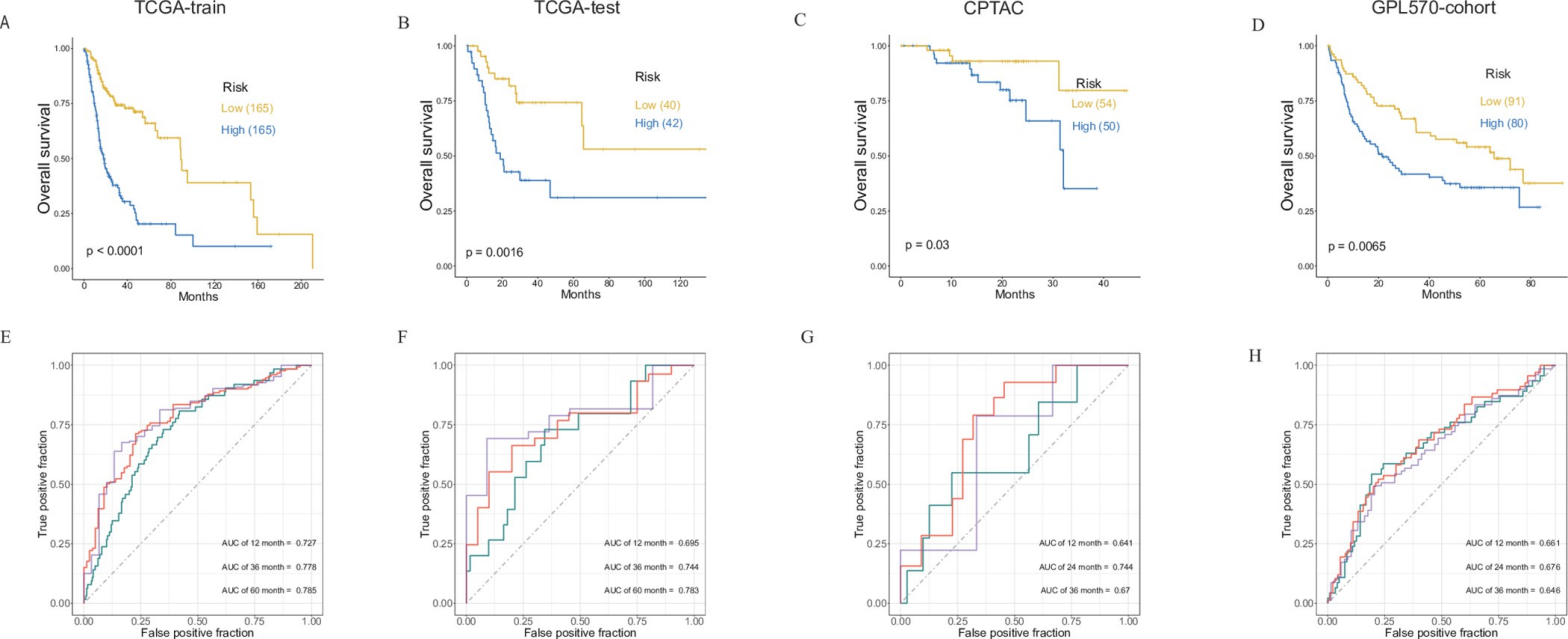
Risk stratification and evaluation of model’s efficacy in HPV-negative HNSCCs. (A-D). Kaplan-Meier survival curves with the log-rank test were performed using the TCGA training set, TCGA internal testing set, and two external validation sets (CPTAC cohort and combined GPL570 cohort) respectively. All samples were classified into different risk groups based on a uniform cutoff value. (E-H). ROC curves were depicted for prognostic signatures in all the datasets.

The prognostic signature was validated using other independent cohorts (**Fig 7**), including TCGA internal testing set, the CPTAC cohort, and the GPL570 combined cohort. Cyclescore efficiently segregated the patients into different risk groups, indicating that the cutoff number can be commonly used in other datasets (**Fig 7A–7D**). The median score of the TCGA training set (-0.0267) served as a cutoff value to segregate the patients into high and low-risk groups for all the tested datasets. AUC values indicated a good efficiency of this analysis in the independent cohorts (**Fig 7E–7H**).

## Discussion

A favorable prognosis was reported in HPV-positive HNSCC patients compared to HPV-negative HNSCCs. Therefore, HPV-positive HNSCC patients were recommended to be treated with lower doses of radiation therapy and chemotherapy [27]. Limited reports investigated the molecular mechanisms associated with a better prognosis for HPV-negative HNSCC patients [27]. Considering the research gap pertinent to HPV-negative HNSCCs, we used database analysis and searched for relevant gene signatures. TCGA database was selected as a relevant database for the analysis as it contains information about gene copy number alterations, gene expression, and mutational profiles, miRNA profiles for more than 520 human HNSCCs [43]. Notably, we focused on the specific genes involved in the regulation of cell cycle progression, including tumor suppressor (TP53) and retinoblastoma protein (RB1). RB1 regulates G1 and S phases of the cancer cell cycle and interacts with the E2F transcription factor [27, 44]. Frequent mutations were observed in tumor suppressor genes (TSG), such as CDKN2A and TP53 in HNSCCs. TP53’s mutation rate in C2 was higher than C1 (83% vs 70%, fisher test P = 0.0037). This was consistent with previous studies; Murugan et al. found that HNSCC with TP53 mutation resulted in a higher cell cycle rate, worse pathological differentiation and more invasive clinical phenotype [45]. HNSCC-associated mutations were found predominantly accumulated in the previously defined 11 genes [43]. One of them, PIK3CA (a catalytic subunit of PI3K, an oncogene kinase) is the most randomly mutated gene in 14% of HNSCCs [46–48]. A total of 23 SMGs were screened by MutSigCV method which included TP53, PIK3CA, HRAS, FAT1, CDKN2A, NOTCH1, CASP8, NFE2L2. There are three genes in the known RAS family: KRAS, NRAS and HRAS. HRAS is the most important isoform of RAS family in HNSCC. When HRAS is in the active state of GTP binding, HRAS can modulate several downstream signal pathways to promote cell growth and proliferation, such as PI3K/Akt and MAPK pathways [49]. In recent years, RAS gene has become a promising target for novel therapy of HNSCC [50]. Several microRNAs downregulate RAS genes, promoting apoptosis and protecting cells during tumorigenesis and uncontrolled cell proliferation [49, 51]. Unlike mutations in the KRAS and NRAS, HRAS functions on the basis of farnesyltransferase activity, this provides an approach to indirectly target oncogenic RAS subtypes using therapeutic modalities with extensive clinical experience [51]. Tipifarnib treatment against HRAS-mutant head and neck squamous cell carcinomas described an ideal overall response rate and survival benefit HNSCCC patients with metastatic HRAS mutations [52]. In addition to the gene mutation, we reported Linc-ROR genetic variants which can also play a vital role in the progression of oral squamous cell carcinoma (OSCC), which needs further research studies.

In the current study, we classified HPV-negative HNSCCs into two subgroups, C1 and C2, according to the identified molecular characteristics, including gene expression patterns of tumor suppressor proteins involved in the regulation of the cancer cell cycle. Molecular patterns and mutational profiles were analyzed in association with clinicopathological characteristics, TME, and responses to immunotherapy and anti-EGFR therapy. We detected significant differences between the C1 and C2 HNSCC subtypes. TP53, DNAH5, NSD1, and RYR2 genes were more frequently mutated in C2; whereas, CASP8 was more frequently mutated in C1. The difference in mutation profiles is correlated with the prognosis, clinical characteristics, TME, and drug efficacy in HPV-negative HNSCCs. These findings are supported by the previously reported analysis [11]. Accordingly, significant clinical responses were observed in some patients with recurrent metastatic HNSCC treated with immune checkpoint inhibitors (FDA-approved agents). A recent study indicated good responses to pembrolizumab (IgG4 antibody the PD-1 receptor) in HNSCC patients [11]. Other PD1 or PDL1 blockers, including ipilimumab (anti-CTLA4 monoclonal antibody), which can regulate adaptive immune functions were shown to stop cancer progression [53]. However, accurate predictive biomarkers are required to guide drug therapy.

Cell cycle progression is often uncontrollable in many tumor types. Abnormal cell division results in enhanced proliferation and cancer metastasis. Recently, clinical trials with cell cycle inhibitors demonstrated gratifying progress, especially in preventing the progression of breast cancers. Palbociclib has been approved for treating HR(+)/HER2(-) advanced breast cancer in China [18–20]. Unexpectedly, the targeted inhibition of the cell cycle in cancer cells was found to promote antitumor immunity [54]. The effect was observed in breast and pancreatic cancers, and melanomas [55–57].

In this study, we identified that HNSCC subtypes can be distinguished using the activation of cell cycle-related genes. Cytotoxic immunocytes infiltration (CD8 T cells, activated NK cells, M1 macrophages, and neutrophils) was higher in subgroup C1, compared to C2. To explore the relationship between the subtypes and intracellular/extracellular TME characteristics, we assessed the correlations using new scoring system generated with Cyclescore. The derived NTP classifier can be used as a new tool to guide the treatment of HPV-negative HNSCC patients. The subtype C1 demonstrated minimal cell cycle activity but a higher sensitivity to anti-PD1 therapy and cetuximab. Our data confirm that cell cycle activity may be linked to the therapeutic effects of anti-PD1 monoclonal antibodies and cetuximab. However, the constructed scoring system warrants further studies to test as a prognostic signature in other independent cohorts.

We observed a significant difference between C1 and C2 subtype characteristics, including the pathological grade and lymph node staging in multiple independent cohorts. However, we were not able to compare clinical parameters exactly as there was insufficient clinical information about TCGA and CPTAC cohort patients. Using the NTP classifier based on cell cycle, HNSCC patients were grouped and indicated differences in the cell cycle pathway activities.

Following this, we explored the application of this subtype for the characterization of TME and treatment-related responses. Using the IOBR package, we detected a positive correlation between several biological processes, including glycolysis, hypoxia, and the presence of myeloid-derived suppressor cells (MDSC). Negative correlations were also observed for activities of KRAS, bile acid metabolism, T cell infiltration, dendritic cells (DC), and B cells. This correlation analysis confirmed the impact of several signaling pathways on the cell cycle initiation/activity, TME, and immune responses.

Among the 23 genes (some of them are described below) included in Cyclescore, some of them were found to be involved in tumor progression and were shown to change TME. For instance, HNSCC proliferation and migration can be inhibited by ENDOU (also known as Placental Protein11 (PP11)) [58]. Another chosen gene CCDC169 is located in cytoband 13q13.3 and correlated with chromosomal aneuploidy in colorectal cancer cells [59]. KRT18 gene was involved in the regulation of metastasis detected in 47 lymph nodes and 48 bone tissues from HNSCC samples [60]. Nuclear factor interleukin 3 (Nfil3) influenced the stability of Treg cells and impaired their immunosuppressive function in vitro experiments [61]. Dead box helicase 41 (DDX41) was also associated with the development of genomic instability and immune abnormalities during myelodysplastic syndrome and acute myeloid leukemia [62]. Consequently, the reported links support the choice of genes that were used for the generation of prognosis signature. However, future experimental studies are warranted to confirm the role of these genes in vivo and uncover the underlying molecular mechanism responsible for HNSCC progression.

## Conclusions

HNSCC is a heterogenous malignancy associated with different cell phenotypes and distinct biological behaviors. Targeted HNSCC treatment strategies are often employed to extend overall survival and quality of life, although deliver poor outcomes. To identify a new prognostic marker, we classified HPV-negative HNSCCs using gene expression patterns of cell cycle-related genes. Employed analytical methods allowed us to group HNSCCs into two subtypes C1 and C2 (with active and inactive cell cycle patterns). C1 differed from C2 in several key markers, including cell cycle activity, tumor stage, mutation profile, prognosis, and responses to immune- and anti-EGFR therapies. Data analysis allowed to the generation of a 23-gene-based prognostic signature, which can be used for the risk classification/assessment of HNSCC patients. We developed and validated an NTP classifier, which helps to predict HNSCC molecular subtypes and guide personalized medicine regimens for suitable patients. The current study provides insights for molecular oncologists and clinicians involved in the assessment and treatment of patients with HNSCC. Our results provide a background for the development of multimodal and personalized HNSCC therapeutic regimens.

## Supporting information

S1 Fig (A) The consensus score matrix; k = 2. (B) CDF presents a real random variable of its probability distribution, analyzed using consensus scores for different cluster numbers (k = 2–9). (C) NbClust was used to explore 26 different criteria and generate the optimal number of clusters. (D, E, F) The PCA, T-SNE and survival analysis of CPTAC cohort based on the results of NTP prediction.
(PDF)

S2 Fig (A). Heatmap of gene expression profiles involved in NTP classifier in combined GPL570 cohort. (B)The survival analysis of combined GPL570 cohort based on NTP prediction. (C-E) Percentage distribution of C1 and C2 subtypes according to tumor stage, pathological N stage and T stage in GSE42743 cohort. (F) Percentage distribution of C1 and C2 subtypes based on tumor stage in GSE142083 cohort.
(PDF)

S3 Fig **Overview of the mutation landscape of the TCGA cohort. (A, B)**. The top 20 genes with the highest mutation frequency of C1 and C2 were showed respectively. **(C)**. Oncoprint for SMGs identified by MutSigCV shown depicted significantly differentially mutated genes based on two subtypes.
(PDF)

S4 Fig The fraction of tumor-infiltrating immune cells among two subtypes in TCGA cohort (****P <0.0001, ***P < 0.001, **P < 0.01, *P < 0.05, ns- not significant). (PDF)

S1 Table Clinical information of 687 HPV-negative head and neck squamous cell carcinoma patients (TCGA-HNSC). (XLSX)

S2 Table A total of 354 The cell cycle related genes used to perform consensus clustering. (XLSX)

S3 Table The 100-gene NTP classifier. (XLSX)

S4 Table The lasso coefficients of corresponding genes in the prognostic signature. (XLSX)
