# Supplementary figures and images for: Molecular classification of human papilloma virus-negative head and neck squamous cell carcinomas: Cell cycle-based classifier and prognostic signature
Source: PLoS One. 2023 Oct 30;18(10):e0286414. doi: 10.1371/journal.pone.0286414 (PMC10615317; doi:10.1371/journal.pone.0286414)

A

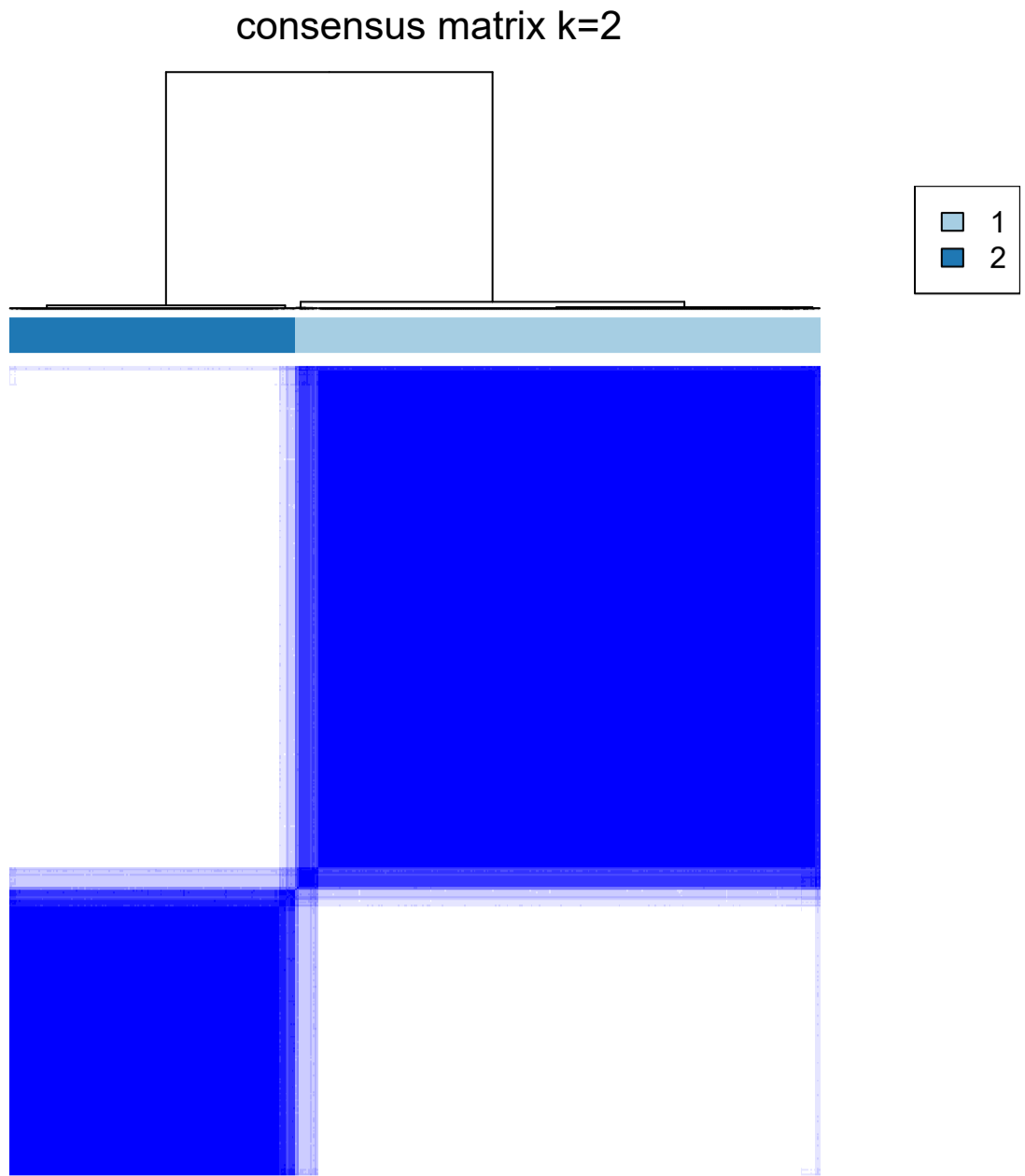

B

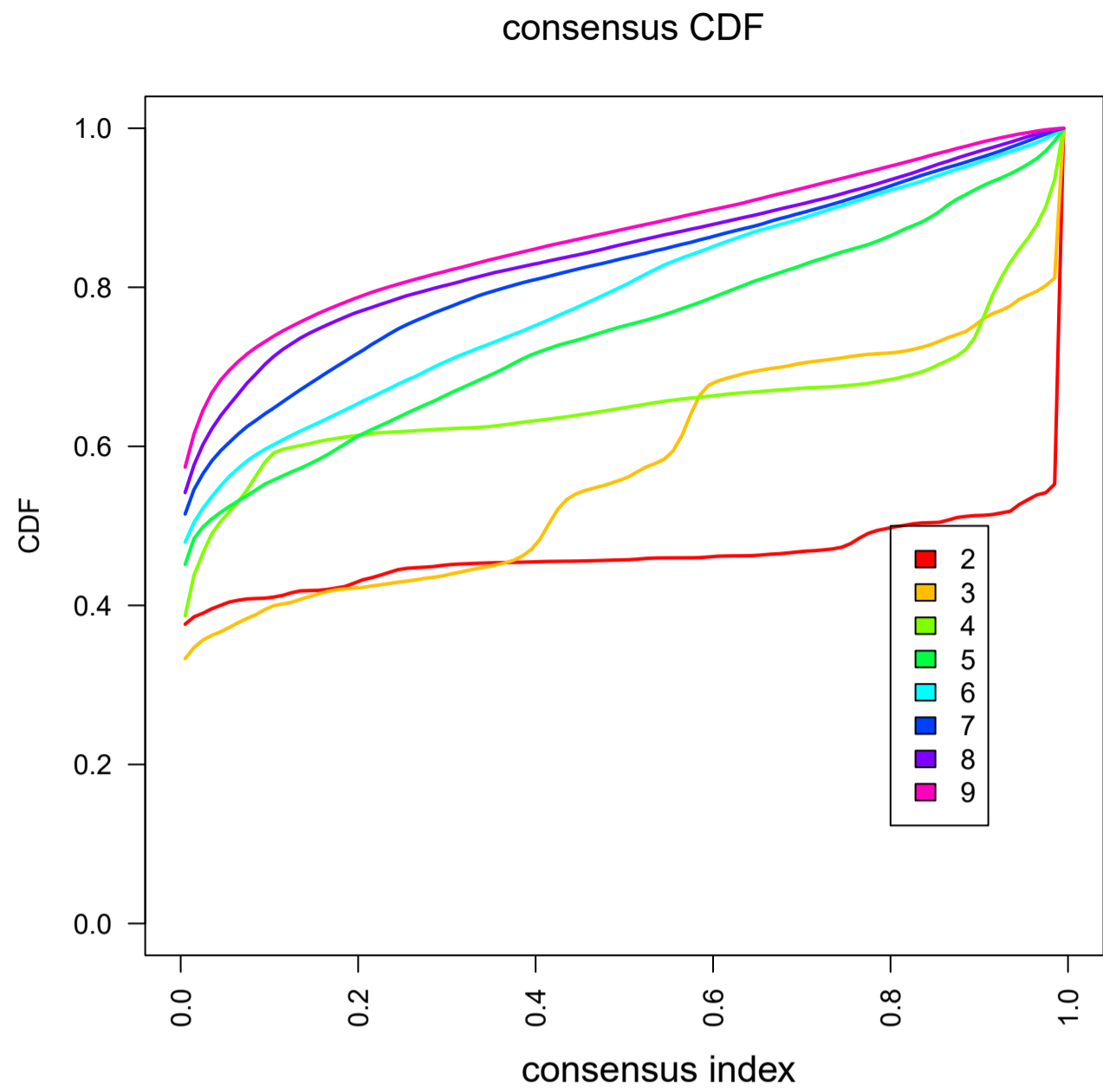

C

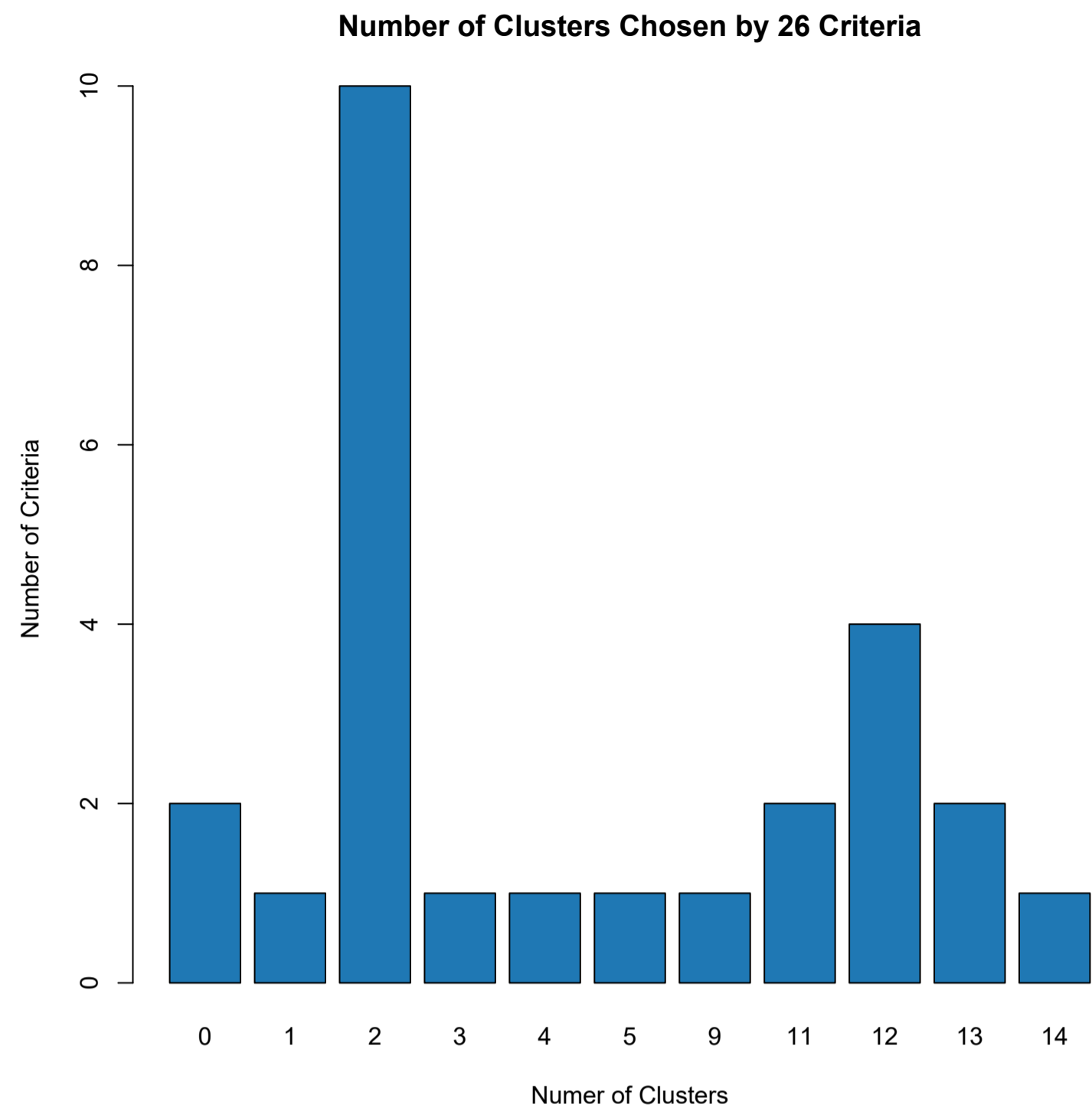

D

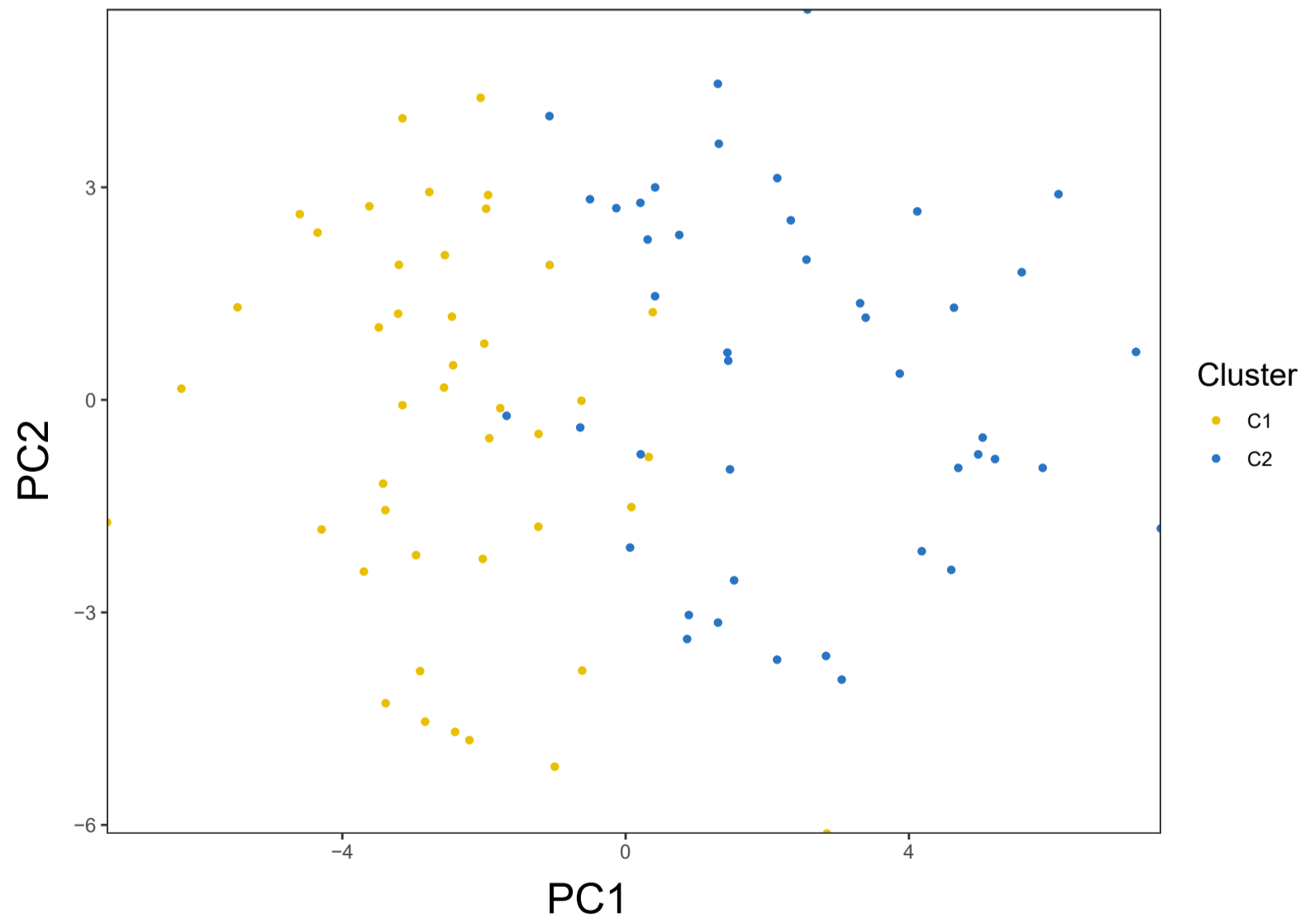

E

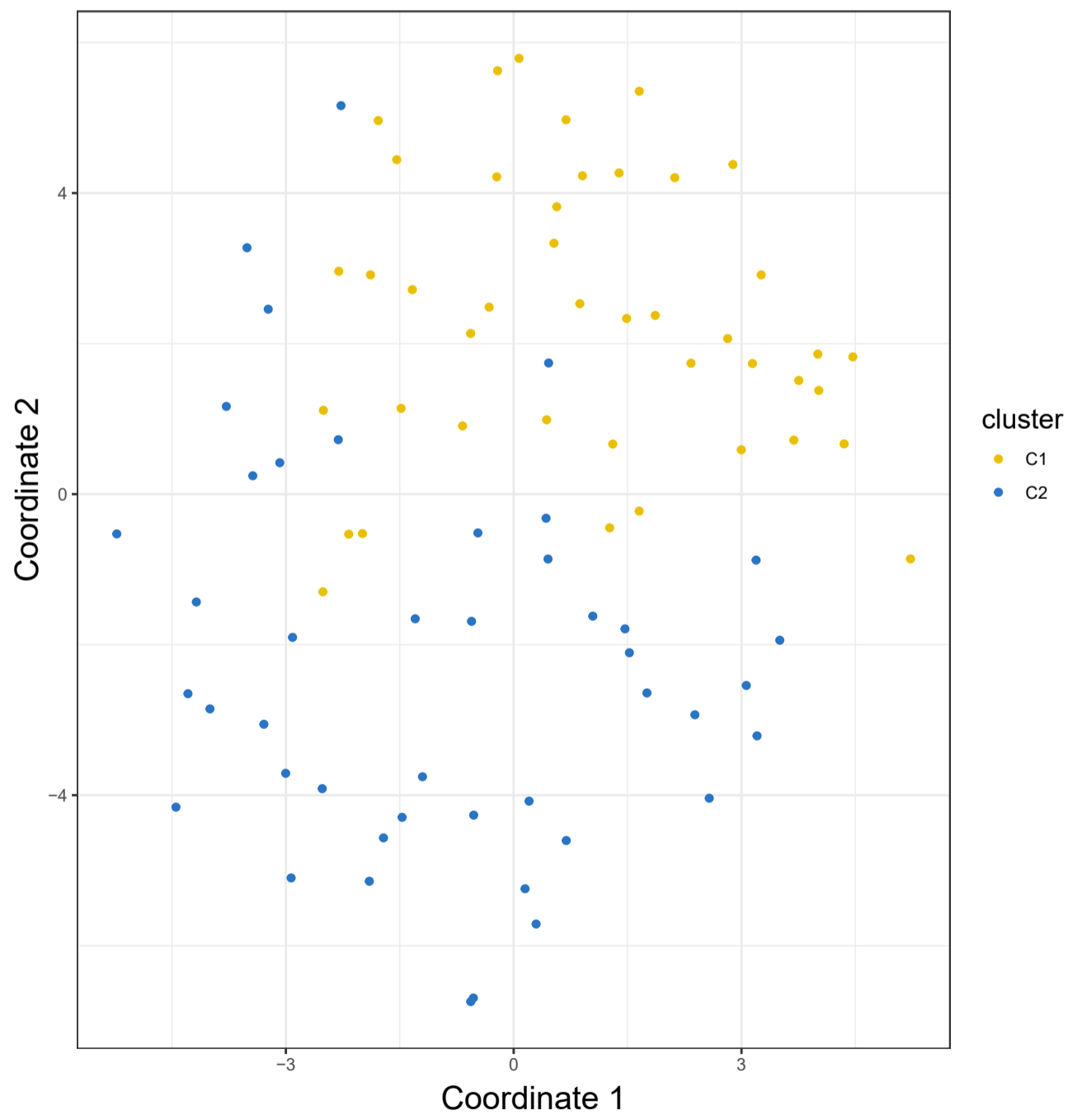

F

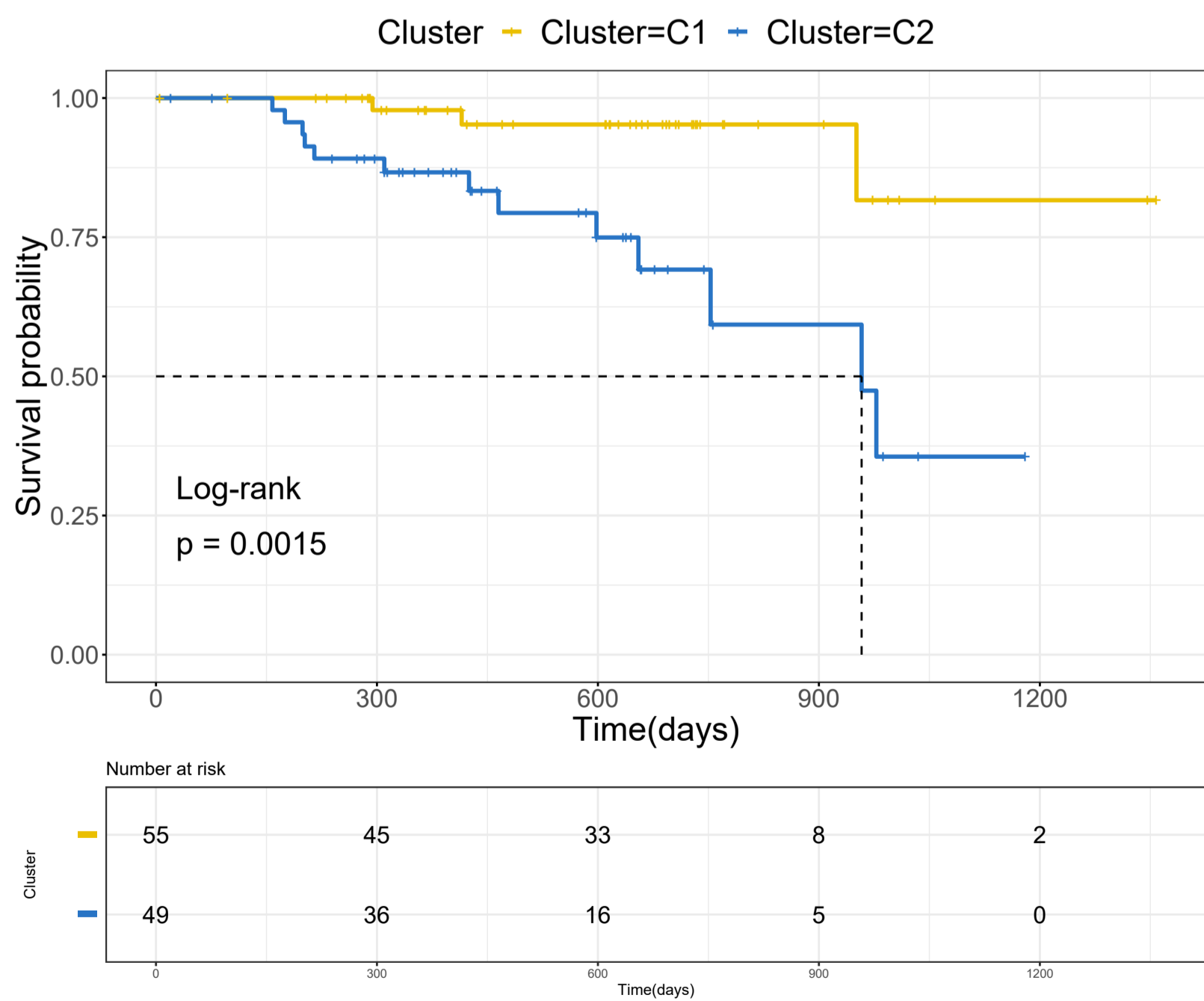

Supplement: S1 Fig — (A) The consensus score matrix; k = 2. (B) CDF presents a real random variable of its probability distribution, analyzed using consensus scores for different cluster numbers (k = 2–9). (C) NbClust was used to explore 26 different criteria and generate the optimal number of clusters. (D, E, F) The PCA, T-SNE and survival analysis of CPTAC cohort based on the results of NTP prediction. (PDF) [file pone.0286414.s001.pdf]

A

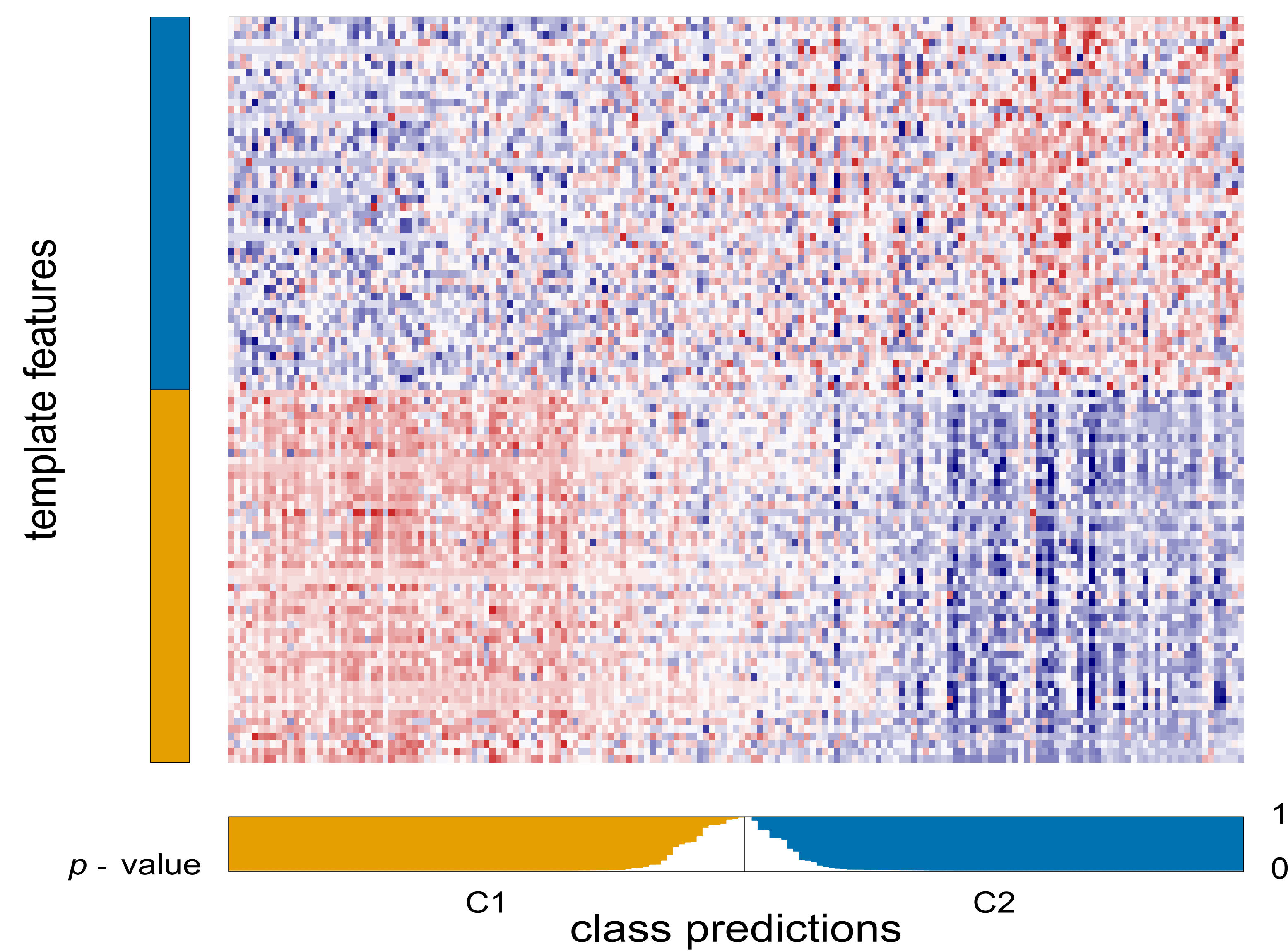

B

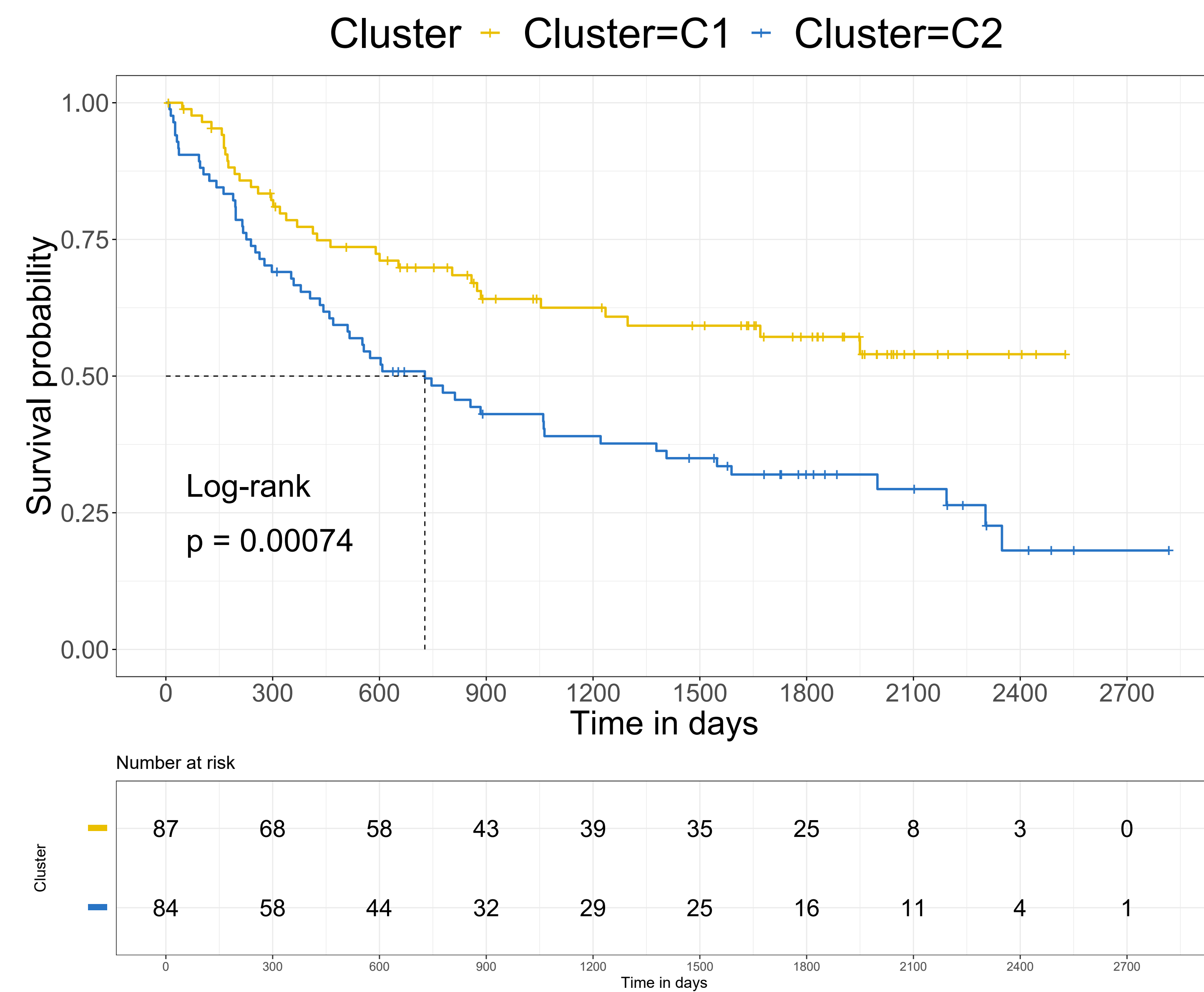

C

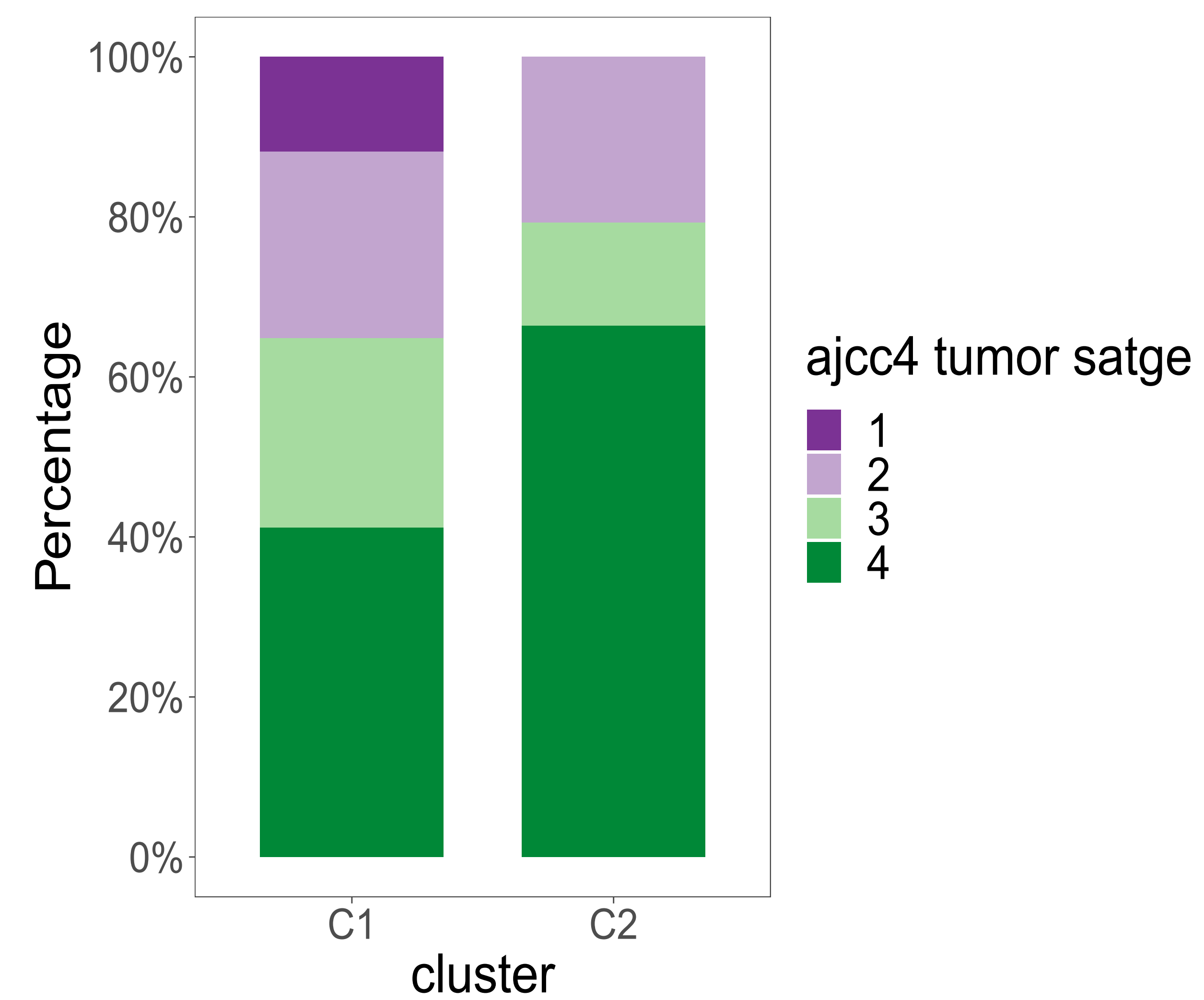

D

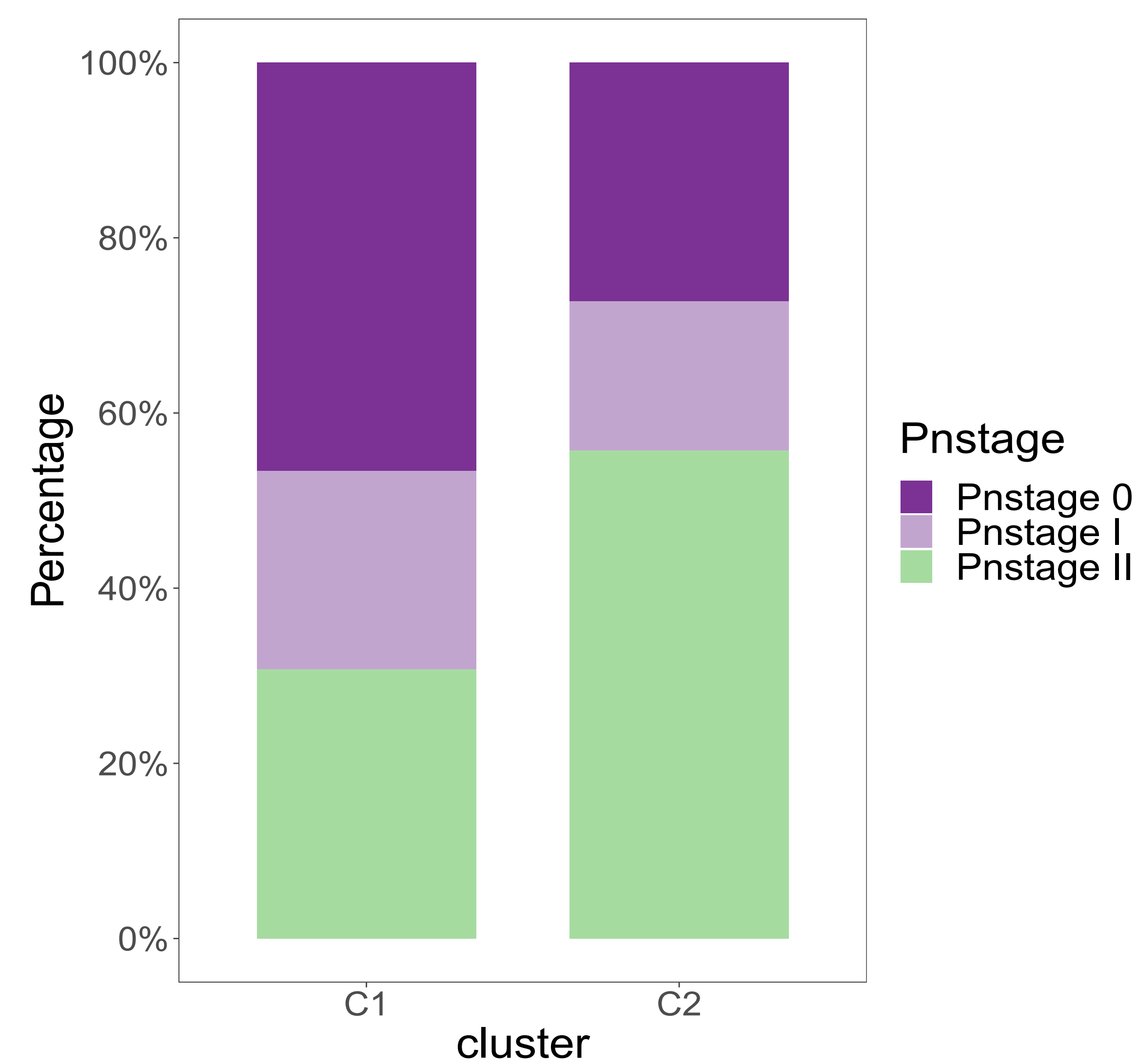

E

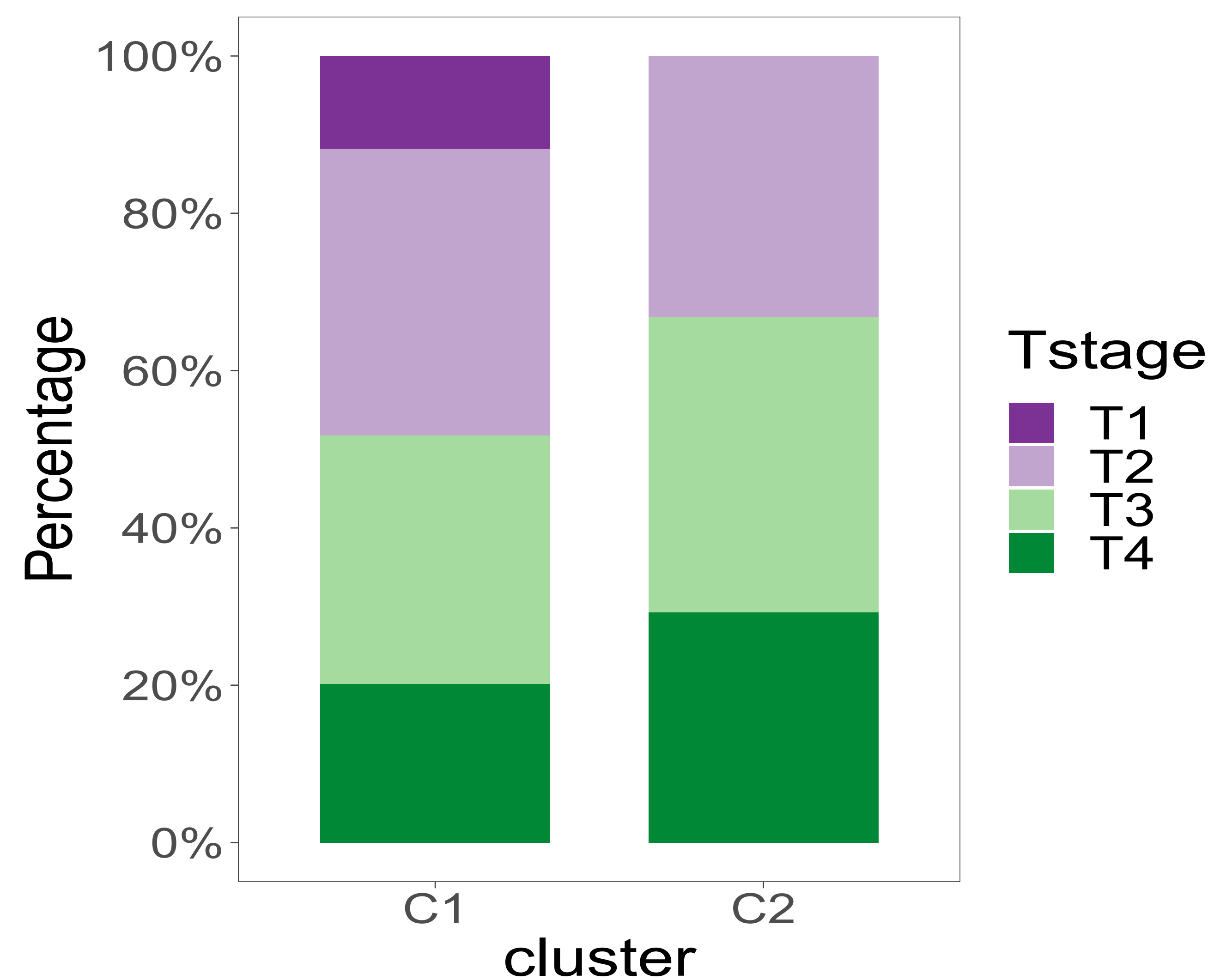

F

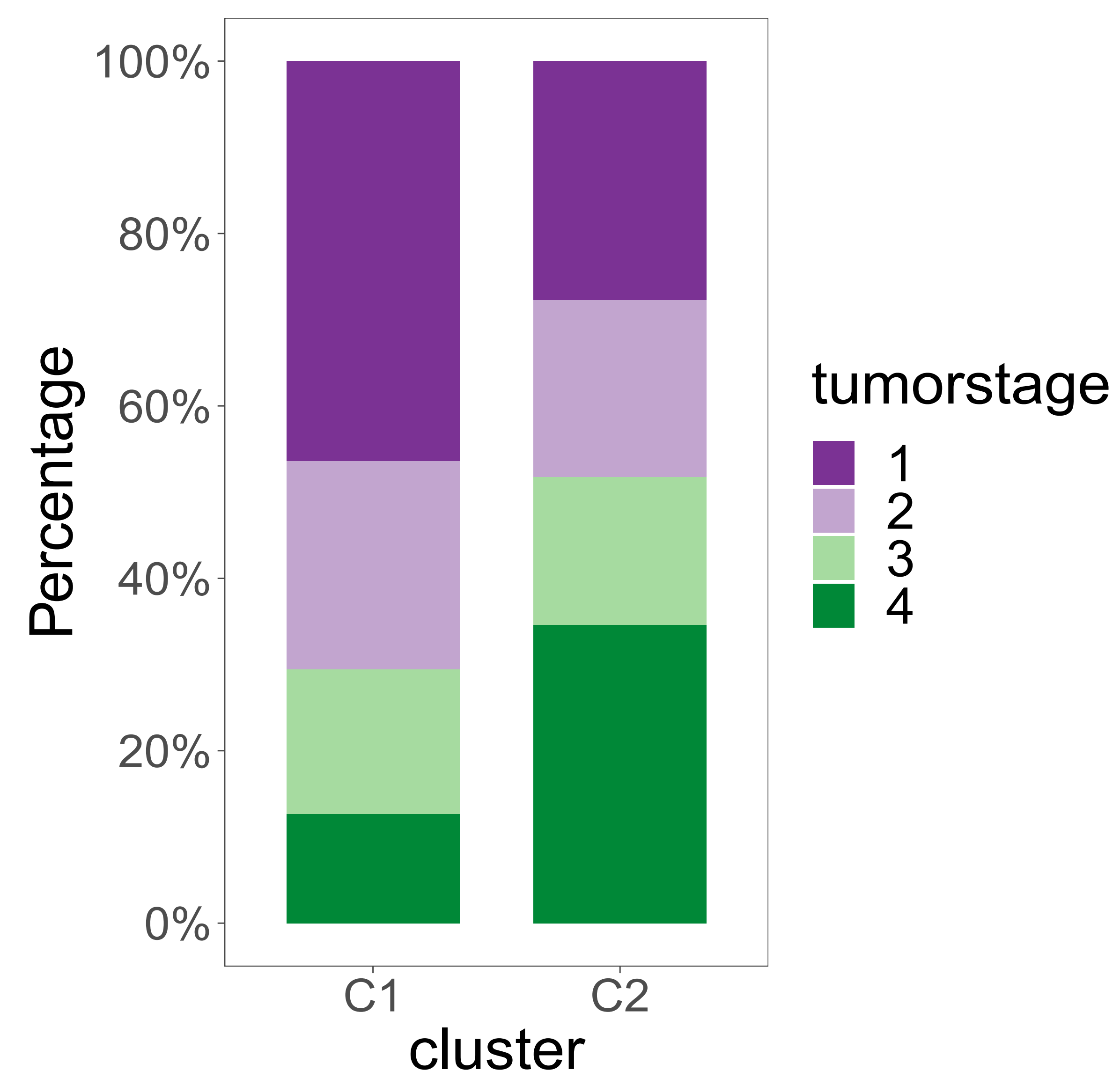

Supplement: S2 Fig — (A). Heatmap of gene expression profiles involved in NTP classifier in combined GPL570 cohort. (B)The survival analysis of combined GPL570 cohort based on NTP prediction. (C-E) Percentage distribution of C1 and C2 subtypes according to tumor stage, pathological N stage and T stage in GSE42743 cohort. (F) Percentage distribution of C1 and C2 subtypes based on tumor stage in GSE142083 cohort. (PDF) [file pone.0286414.s002.pdf]

A

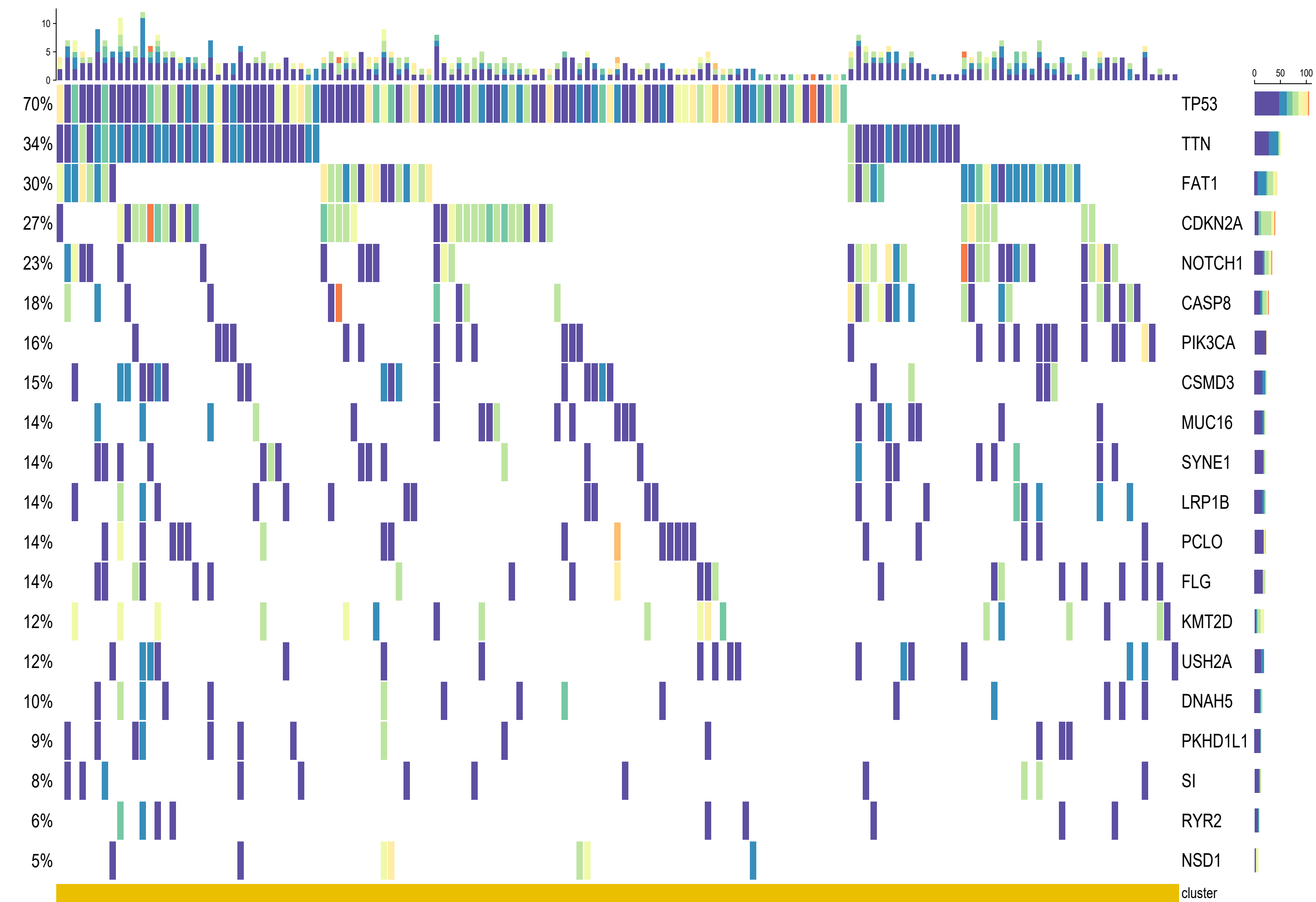

B

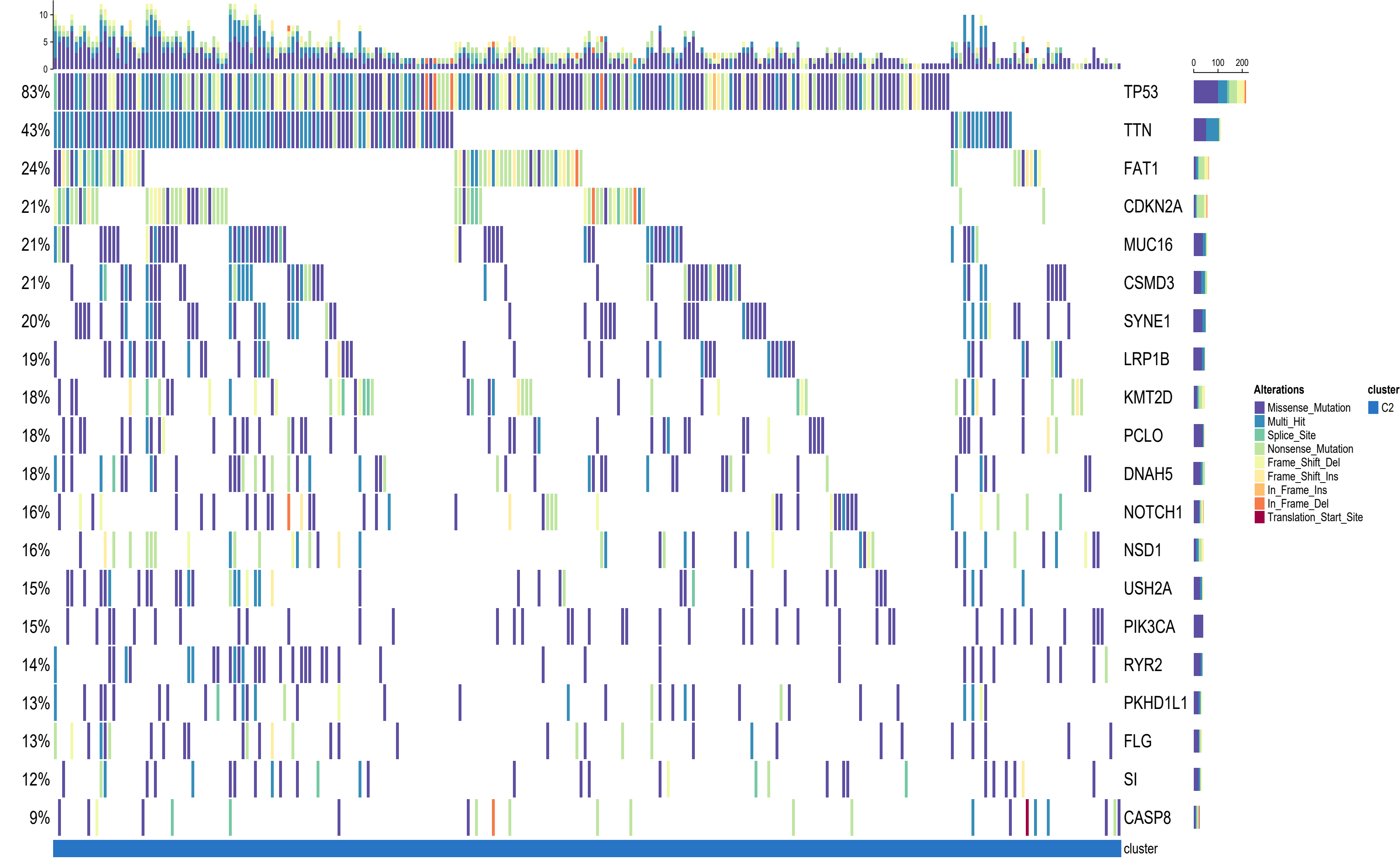

C

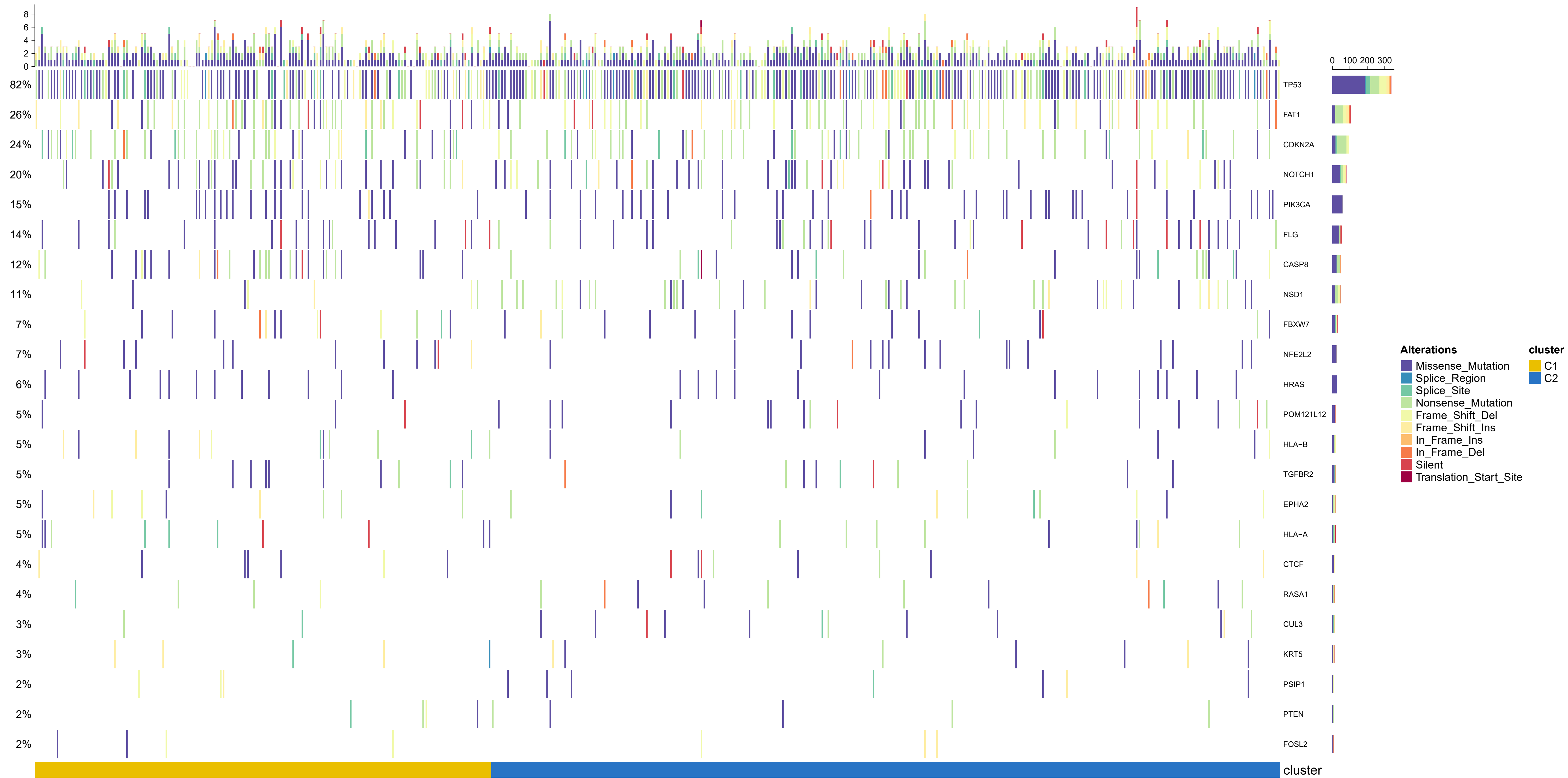

Supplement: S3 Fig — Overview of the mutation landscape of the TCGA cohort. (A, B). The top 20 genes with the highest mutation frequency of C1 and C2 were showed respectively. (C). Oncoprint for SMGs identified by MutSigCV shown depicted significantly differentially mutated genes based on two subtypes. (PDF) [file pone.0286414.s003.pdf]

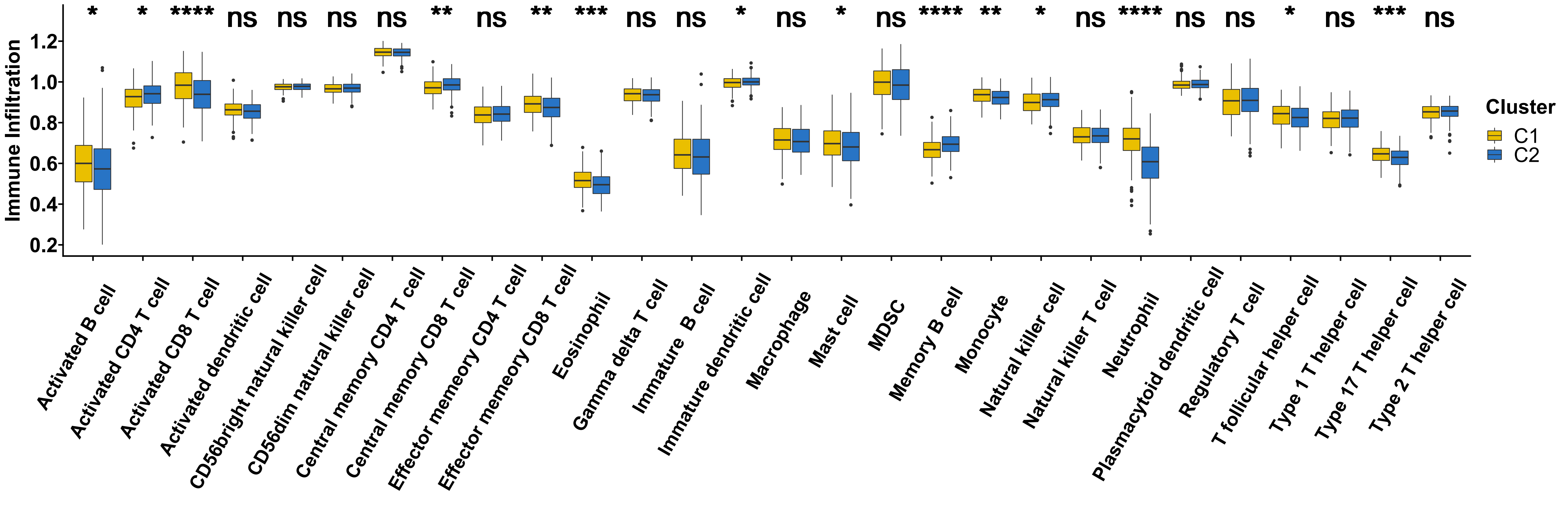

Supplement: S4 Fig — (PDF) [file pone.0286414.s004.pdf]
